# Targeting Aggrecanases for Osteoarthritis Therapy: From Zinc Chelation to Exosite Inhibition

**DOI:** 10.1021/acs.jmedchem.2c01177

**Published:** 2022-10-17

**Authors:** Doretta Cuffaro, Lidia Ciccone, Armando Rossello, Elisa Nuti, Salvatore Santamaria

**Affiliations:** † Department of Pharmacy, University of Pisa, via Bonanno 6, 56126 Pisa, Italy; ‡ Department of Immunology and Inflammation, Imperial College London, Du Cane Road, London W12 0NN, U.K.

## Abstract

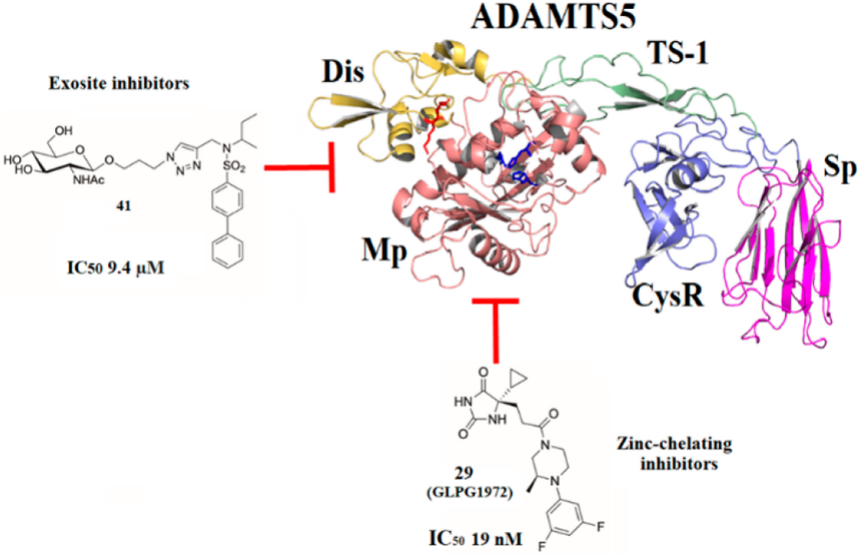

Osteoarthritis (OA) is the most common degenerative joint disease. In 1999, two members of the A Disintegrin and Metalloproteinase with Thrombospondin Motifs (ADAMTS) family of metalloproteinases, ADAMTS4 and ADAMTS5, or aggrecanases, were identified as the enzymes responsible for aggrecan degradation in cartilage. The first aggrecanase inhibitors targeted the active site by chelation of the catalytic zinc ion. Due to the generally disappointing performance of zinc-chelating inhibitors in preclinical and clinical studies, inhibition strategies tried to move away from the active-site zinc in order to improve selectivity. Exosite inhibitors bind to proteoglycan-binding residues present on the aggrecanase ancillary domains (called exosites). While exosite inhibitors are generally more selective than zinc-chelating inhibitors, they are still far from fulfilling their potential, partly due to a lack of structural and functional data on aggrecanase exosites. Filling this gap will inform the design of novel potent, selective aggrecanase inhibitors.

## 1 Introduction

### 1.1 Aggrecanases as Targets in OA

Osteoarthritis (OA) is the most common chronic degenerative joint disease, representing a leading cause of years lived with disability worldwide.^1^ This places a large socio-economic burden on healthcare systems, with estimated medical costs between 1 and 2.5% of the gross domestic product in high-income countries.^2^ OA affects predominantly the knee, hip, and hand joints.^1,3^ In severely affected OA patients, joint replacement surgery is the only viable option, although is not a risk-free option.^4^ Pharmacological treatment for symptomatic OA is largely palliative, being limited to steroidal and non-steroidal anti-inflammatory drugs (NSAIDs), which are unable to alter disease progression.^5^ NSAIDs have also raised safety concerns, especially considering long-term administration on an aged population with multiple co-morbidities such as cardiovascular diseases, diabetes, and obesity.^6^ No drugs able to slow down or halt the progression of OA, i.e., disease-modifying OA drugs (DMOADs), are currently available, and this led the U.S. Food and Drug Administration (FDA) in 2018 to label OA as a “serious disease with an unmet medical need”.^7^ This is not the result of a lack of efforts from pharmaceutical companies and academic institutions—quite the contrary.

OA is a complex multifactorial disease whose pathogenetic mechanisms are still not completely understood. Some promising DMOADs under development target cartilage degradation, a major hallmark of OA.^6,8,9^ Since articular cartilage allows for low-friction movement between bones, its erosion is a major cause of impaired mobility and pain. Articular cartilage is composed by chondrocytes embedded in an extracellular matrix (ECM) rich in collagens (of which types II, VI, and XII are the most abundant) and proteoglycans such as aggrecan and, in low amounts, biglycan.^10^ Collagens provide the tissue with tensile strength, whereas aggrecan provides compressibility through its ability to regulate osmotic pressure via the Donnan effect.^11,12^ This function of aggrecan is mediated by the negatively charged glycosaminoglycan (GAG) chains attached to its protein core, which attract counterions from the interstitial fluid filling the cartilage pores. Not surprisingly, net loss of both collagens and aggrecan has a devastating effect on cartilage integrity, the latter representing an early, reversible phase of the dysregulated ECM catabolism which is typical of OA.^13,14^

Perhaps because of the early failure of collagenase inhibitors in cancer clinical trials,^15^ exploration of this class of molecules as DMOADs has been limited. Poor selectivity, lack of efficacy, and musculoskeletal (MSK) adverse effects such as joint stiffness and pain hampered further applications of matrix metalloproteinase inhibitors (MMPs), the class of ECM proteases endowed with collagenase activity. For example, a phase II clinical trial for knee OA with the MMP inhibitor PG-116800, developed by Procter & Gamble, was terminated due to an increased frequency of adverse MSK effects such as arthralgia (ClinicalTrials.gov Identifier: NCT00041756).^16^

At a time when research on collagenase inhibitors was stalling, two distinct aggrecanase activities were isolated and identified as members of A Disintegrin and Metalloproteinase with Thrombospondin Motifs (ADAMTS) family of metalloproteinases: aggrecanase-1 (ADAMTS4)^17^ and aggrecanase-2 (ADAMTS5, originally named ADAMTS11).^18^ Since then, four lines of evidence have supported the choice of ADAMTS5 as a favored target in OA:^19^ (1) ADAMTS5 is the most potent proteoglycanase *in vitro*;^20−22^ (2) in contrast with *Adamts4* knockout mice,^23^*Adamts5* knockout mice showed protection in inflammatory or surgical OA models;^24,25^ (3) anti-ADAMTS5 monoclonal antibodies (mAbs) effectively inhibited aggrecan degradation in human *ex vivo* OA models;^26−28^ (4) ADAMTS5 accumulation is sufficient to lead to aggrecan degradation in human chondrocyte monolayer cultures.^29^ Both genetic ablation^30^ and selective inhibition^31^ of ADAMTS5 in mice also reduced OA-related pain sensitization (allodynia); thus, ADAMTS5 inhibitors may show additional analgesic effects.

Notwithstanding the prominent role of ADAMTS5 in OA pathology, simultaneous inhibition of ADAMTS4 may not be undesirable as an OA treatment, given that ADAMTS4 expression is consistently upregulated under inflammatory conditions.^19,32^ Provided that both side and off-target effects are carefully evaluated, inhibitors targeting both aggrecanases may exhibit a competitive advantage over those selectively directed against just one of them.

**Figure 1:**
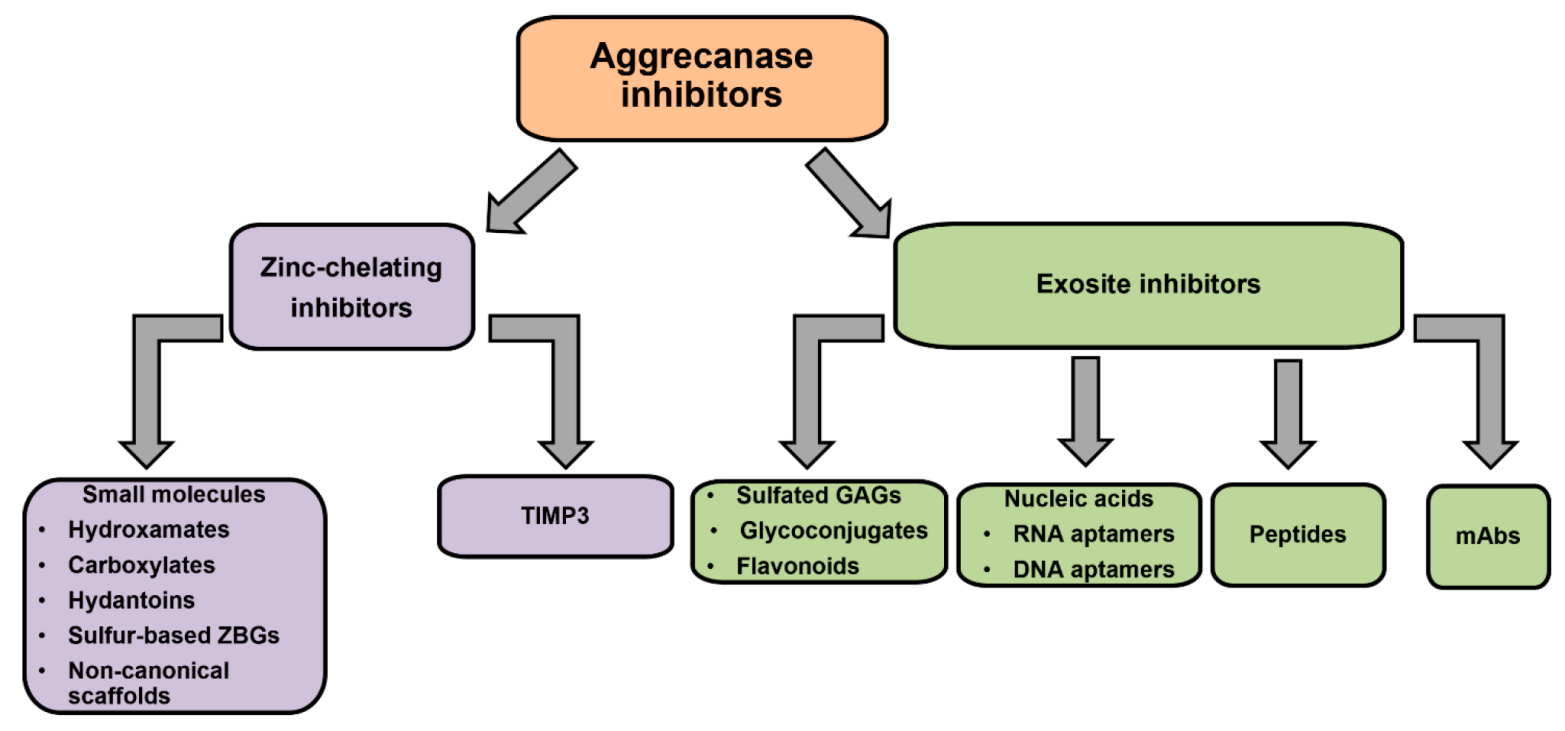
Classification of aggrecanase inhibitors.

Aggrecanase inhibitors can be classified in two groups on the basis of their mechanism of inhibition, i.e., zinc-chelating inhibitors and exosite inhibitors (Figure 1). Zinc-chelating inhibitors comprise mostly synthetic, low-molecular-weight molecules as well as the endogenous aggrecanase inhibitor Tissue Inhibitor of Metalloproteinase 3 (TIMP3) and its engineered variants. Exosite inhibitors interact with non-catalytic residues involved in substrate recognition and cleavage, i.e., exosites. These are defined as small clusters of non-adjacent residues in the ADAMTS ancillary domains, which are poorly conserved between the different ADAMTS family members.^22^ Exosite inhibitors comprise sulfated GAGs, glycoconjugates, flavonoids, nucleic acids, peptides, and monoclonal antibodies (mAbs). Because of their ability to target non-conserved residues, exosite inhibitors are expected to be more selective than zinc-chelating inhibitors.

A comparison of zinc-chelating versus exosite inhibitors is instrumental in informing the development of potent, selective aggrecanase inhibitors. Here, we review the current literature on aggrecanase inhibitors as of April 2022. Data were obtained from different sources, including PubMed, the clinical trial database (www.clinicaltrials.gov), patents, company web sites, and abstracts from international congresses. We focus on molecules that act by directly inhibiting ADAMTS4 and -5, while molecules that interfere with their post-transcriptional regulation, such as 2-(8-methoxy-2-methyl-4-oxoquinolin-1(4*H*)-yl)-*N*-(3-methoxyphenyl)acetamide^33^ and small interfering RNAs,^34,35^ are outside the scope of this review. We initially present the data currently available on the structures of ADAMTS4 and ADAMTS5 and then proceed to a detailed comparison of zinc-chelating and exosite inhibitors by highlighting advantages and drawbacks of the two approaches.

### 1.2 Fold and Functions of Aggrecanase Domains

ADAMTS4 and ADAMTS5 belong to family M12 in clan MA of the metallopeptidases. Proteases in clan MA are collectively called “metzincins”, due to the presence of a conserved signature composed of a zinc-chelating sequence (HEXXHXXG/NXXH/D) followed C-terminally by a methionine residue.^36^ Other protease families in clan MA comprise the above-mentioned MMPs and A Disintegrin and Metalloproteinases (ADAMs), the latter including only transmembrane members.

The domain composition of ADAMTS4 and ADAMTS5 consists of a signal peptide, a prodomain, a metalloproteinase catalytic domain (Mp), followed by non-catalytic ancillary domains such as a disintegrin-like (Dis) domain, a central thrombospondin-type I motif (TS-1), a cysteine-rich (CysR) domain, and a spacer (Sp) domain. ADAMTS5 displays an additional TS-1 motif at the C terminus.

Both aggrecanases are expressed as inactive zymogens with a large prodomain (161 and 245 residues in ADAMTS4 and ADAMTS5, respectively) necessary to maintain latency. The mechanism behind the inhibitory function of the prodomain has not been elucidated. In related MMPs, the S^γ^ of a conserved cysteine residue within the sequence PRCGVPD coordinates the active-site zinc,^37^ but it is not known if this “cysteine switch” mechanism is also present in aggrecanases. Both prodomains contain a sequence with low homology (^192^PMCNVKAP^199^ and ^207^ASCETPAS^214^ in ADAMTS4 and ADAMTS5, respectively) to the MMP sequence. Unfortunately, no structure is available for the prodomain of aggrecanases, and AlphaFold predicts the structure of this domain with very low confidence (per-residue confidence score <50; IDs: AF-O75173-F1 and AF-Q9UNA0-F1 for ADAMTS4 and ADAMTS5, respectively). To the best of our knowledge, no mutations of the cysteine residues in the putative cysteine-switch sequences have been reported. ADAMTS4 and ADAMTS5 activation requires proteolytic removal of the prodomain by proprotein convertases such as furin and PACE4,^38−40^ which cleave downstream the multi-basic sequences ^206^RPRRAKR^212^ and ^257^RRRRR^261^ in ADAMTS4 and ADAMTS5, respectively.

**Figure 2:**
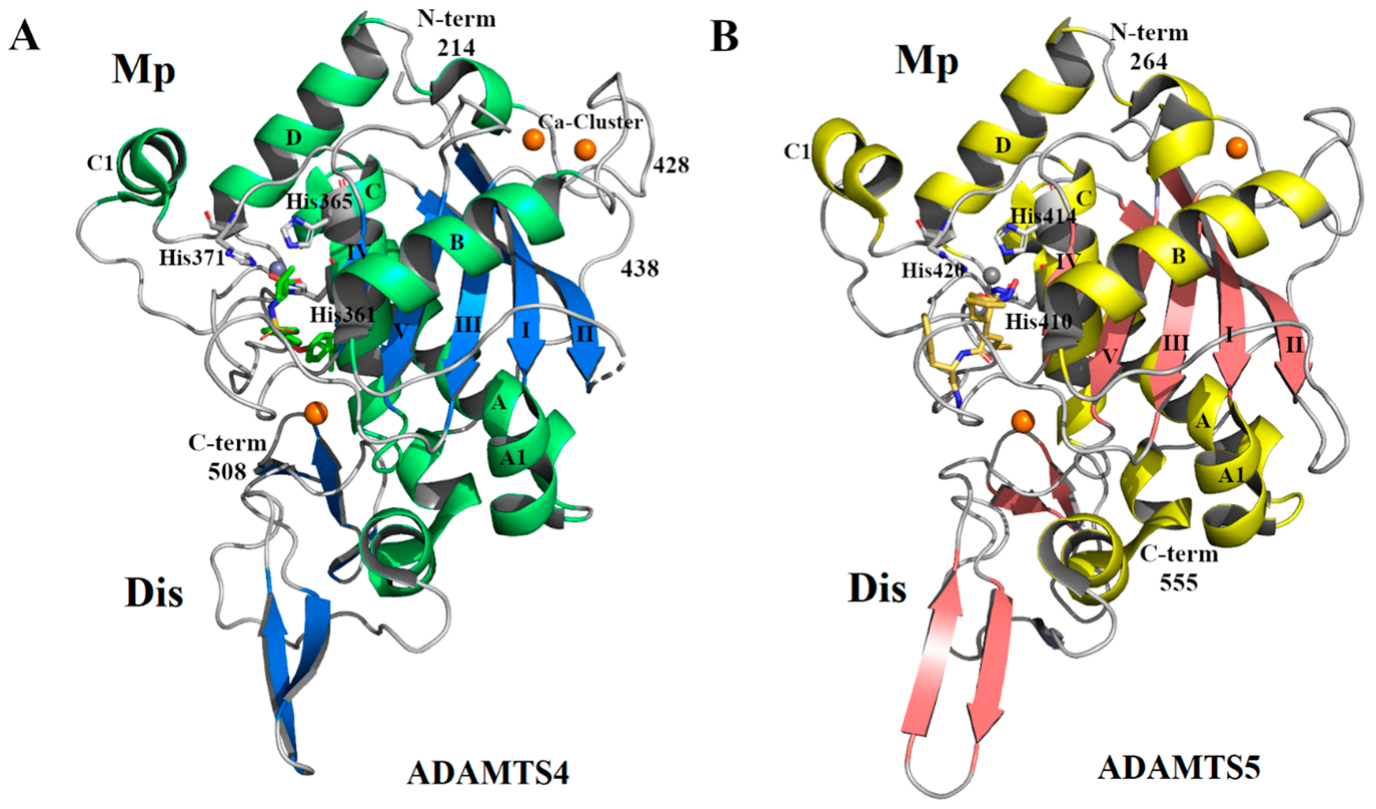
Crystal structure of the Mp/Dis domains of ADAMTS4 (PDB 2RJP) (A) and ADAMTS5 (PDB 2RJQ) (B). In (A) the β-sheets are colored marine while α-helices are colored lime green; in (B) the β-sheets are colored light pink while α-helices are colored pale yellow; the catalytic Zn^2+^ is highlighted in gray and the Ca^2+^ ions in orange. Structures have been generated in PyMol^43^ by modifying previous scripts,^44,45^ and assembled using GNU Image Manipulation Program (GIMP).^46^

Currently, 5 crystal structures have been deposited in the Protein Databank for ADAMTS4 and 7 for ADAMTS5 (Uniprot IDs O75173 and Q9UNA0, respectively), none of them covering regions C-terminal to the Dis domain. The two aggrecanases show a very similar fold across the Mp/Dis domains (Figure 2). The Mp domain (residues 213–428 and 262–476 in ADAMTS4 and ADAMTS5, respectively) is characterized by the α/β structure typical of clan MA, with a central core of five-stranded β-sheet; four long strands are in parallel (I, II, III, and V), and a short fifth (IV) is in antiparallel configuration. The β-sheet is surrounded by α-helices A, A1, B, C, C1, and D. While helices A and C are common to those of other MMP and ADAM structures, helix B is typical of ADAMTS4 and ADAMTS5.^41,42^

**Figure 3:**
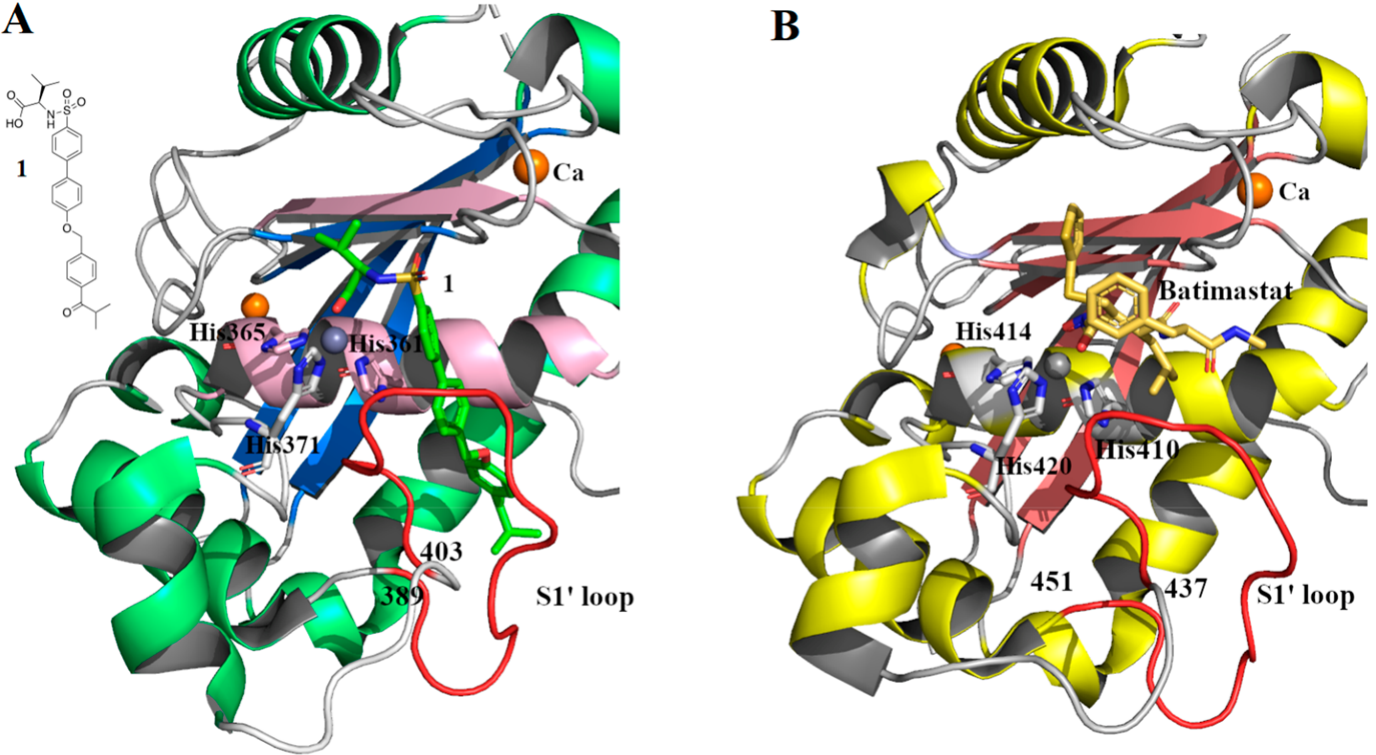
Structure of the aggrecanase Mp domain in complex with hydroxamate inhibitors. (A) Crystal structure of ADAMTS4 (PDB 2RJP) complexed with compound **1**; the β strand and α helix of the active site are shown in light pink, the S1′ loop in red. (B) Crystal structure of ADAMTS5 (PDB 2RJQ) in complex with Batimastat. The S1′ loop is shown in red. Zinc and calcium ion are colored in gray and in orange, respectively.

The Mp domain contains the active site, a cleft parallel to helix C where a catalytic Zn^2+^ ion is coordinated by three conserved His residues (ADAMTS4: His361, His365, His371; ADAMTS5: His410, His414, His420) (Figure 3). In addition to the Zn^2+^ ion, the Mp domain contains two or three Ca^2+^ ions; in ADAMTS4 two Ca^2+^ ions are located in a Ca-cluster flanked by disulfide bridges (Figure 2A). This Ca^2+^-cluster site is another unique aspect of ADAMTSs with respect to MMPs.

When a zinc-binding inhibitor is bound to the active site, ADAMTS4 and ADAMTS5 display a similar shape of the subsites (S1, S1′, S2, S2′, and S3, S3′, according to the Schechter and Berger nomenclature).^47^ A comparison of the two Mp domains with those of MMPs suggests that the major differences are located around the S2′ and S1′ pockets. Compared to MMPs, the S2′ pocket is smaller and characterized by a unique motif sequence (^322^CGVSTCDT^329^ and ^371^CGHHSCDT^378^ for ADAMTS4 and ADAMTS5, respectively). The lipophilic S1′ pocket, formed by the base of strand IV, a part of helix C and an adjacent loop (amino acids 389–403 and 437–451 in ADAMTS4 and ADAMTS5, respectively), is able to assume different conformations based on the inhibitor bound (Figure 3). Even if the active site is highly conserved in the two aggrecanases, the presence of four different residues (Ala252, Val390, Met395, and Val398 in ADAMTS4 compared to Leu301, Leu438, Leu443, and Ile446 in ADAMTS5) leads to a larger S1′ pocket in ADAMTS4. For this reason, inhibitors with bulky P1′ groups usually possess greater inhibitory activity against ADAMTS4 than ADAMTS5 (see section 2).

**Figure 4:**
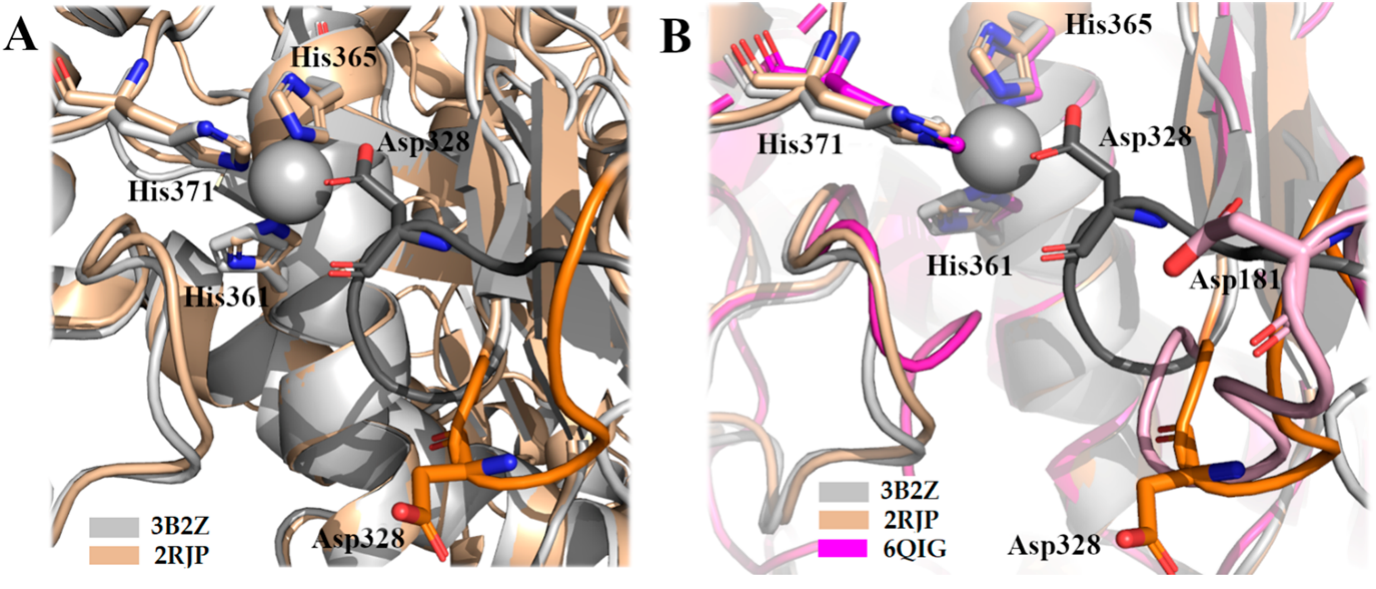
Auto-inhibitory mechanism in ADAMTS4. (A) Superimposition between the Mp domain of uninhibited ADAMTS4 (PDB 3B2Z) and ADAMTS4 in complex with compound **1** (PDB 2RJP). Crystal structures of uninhibited ADAMTS4 is colored gray; the S2′ loop is highlighted in dark gray with the Asp328 chelating Zn^2+^ pointed out in sticks. The Mp domain of ADAMTS4 in complex with inhibitor (ligand not shown) is in brown, and the S2′ loop with Asp328 is highlighted in orange. (B) Comparison between the Mp domain of ADAMTS4 in uninhibited and inhibited forms (PDB 3B2Z and 2RJP) and the Mp domain of ADAMTS13 (PDB 6QIG). The S2′ loop of ADAMTS13, highlighted in light pink, shows a similar conformation to the S2′-loop of inhibited ADAMTS4. Zinc ion is colored in gray.

While the structures of ADAMTS4 and ADAMTS5 in complex with zinc-chelating hydroxamate inhibitors are very similar to each other, the uninhibited form of ADAMTS4 has a different conformation, with the carboxylic group of Asp328 in the S2′ loop coordinating the Zn^2+^ ion (Figure 4A). The global shift of the S2′ loop toward the active site in ADAMTS4 suggests an auto-inhibitory mechanism not present in MMPs, where the active site is wholly exposed in absence of any ligand, or in other known ADAMTS structures. In ADAMTS13, the best characterized ADAMTS family member, a different auto-inhibitory mechanism is in place.^48^ Here, Asp181 in the S2′ loop does not interact with the catalytic zinc (Figure 4B); instead, a non-proteolytically competent conformation is guaranteed by a “gatekeeper triad” of charged residues (Arg193, Asp217, and Asp252) that, through a hydrogen bond network, occlude the catalytic cleft.^48^ A superimposition between the crystal structure of the ADAMTS4 Mp domain in its free (PDB 3B2Z) and inhibited (PDB 2RJP) forms and that of free ADAMTS13 (PDB 6QIG) shows that the conformation of the S2′ loop in the presence of hydroxamate inhibitor **1** is the one that more closely resembles ADAMTS13 (Figure 4B).

Downstream from the zinc-binding sequence, there is also the methionine residue (Met369 and Met439 in ADAMTS4 and ADAMTS5, respectively) of the “Met-turn”, a topological constraint conserved in metzincins which is required for the structural integrity of the zinc-binding site.^49^

**Figure 5:**
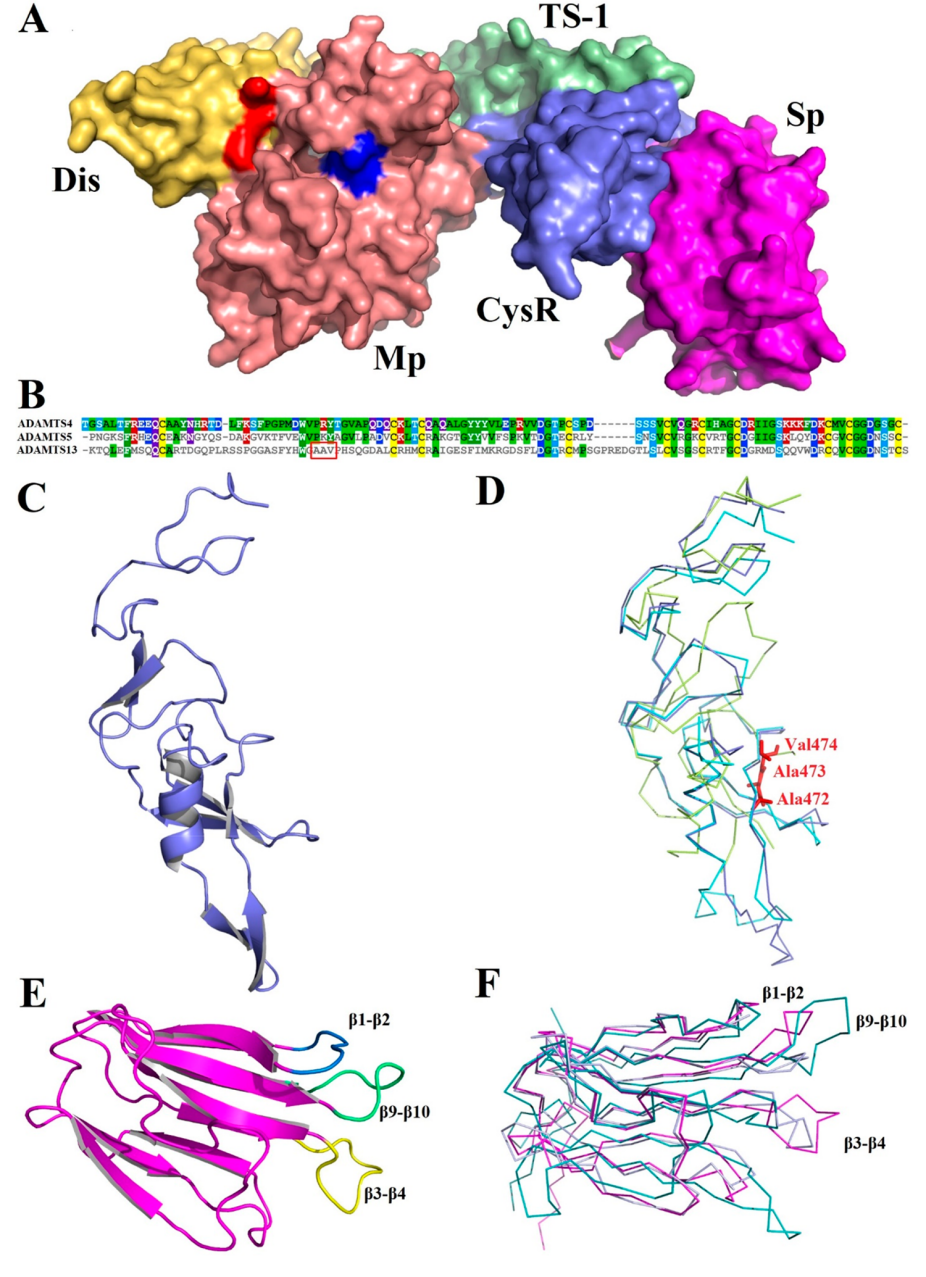
Exosites in ADAMTS5 ancillary domains. (A) Surface structure of ADAMTS5 as predicted by AlphaFold;^54^ each domain is labeled and highlighted with different colors (prodomain and the C-terminal TS-1 domain not shown). The three His residues of the catalytic cleft in the Mp domain are highlighted in blue; the exosite in the Dis domain (^532^KK^533^) is shown in red. (B) Amino acid sequence alignment of the CysR of ADAMTS4 (UniProt ID: O75173, residues 576–685), ADAMTS5 (UniProt ID: Q9UNA0, residues 623–731), and ADAMTS13 (UniProt ID: Q76LX8, residues 440–556). Alignment was performed in ClustalOmega (https://www.ebi.ac.uk/Tools/msa/clustalo/) and visualized using MView (https://www.ebi.ac.uk/Tools/msa/mview/). In ADAMTS13, the vWF-binding exosite ^472^AAV^474^ [**52**] is highlighted. Boxes indicate amino acids conserved in at least two of the three proteases and are colored according both amino acid identity and physicochemical properties (red, positively charged; purple, negatively charged; green, apolar; cyan and pink, polar), while cysteine residues are in yellow. (C) Cartoon model of ADAMTS5 CysR domain (AlphaFold ID: AF-Q9UNA0-F1). (D) Superimposition of the Alphafold model of ADAMTS4 CysR domain (AlphaFold ID: AF-O75173-F1) (cyan), ADAMTS5 (AlphaFold ID: AF-Q9UNA0-F1) (slate), and the crystal structure of ADAMTS13 (PDB 6QIG) (lemon). (E) Cartoon representation of ADAMTS5 Sp domain (AlphaFold ID: AF-Q9UNA0-F1); exosites are highlighted with different colors. (F) Superimposition of the Alphafold model of ADAMTS4 Sp domain (AlphaFold ID: AF-O75173-F1) (slate), ADAMTS5 (AlphaFold ID: AF-Q9UNA0-F1) (magenta), and crystal structure of ADAMTS13 (PDB 6QIG) (teal).

In both ADAMTS4 and ADAMTS5, the Dis domain adopts a common fold characterized by two α-helixes and two β-sheets connected by several loops (Figure 2). Despite its name, this region shows no structural homology to the Dis domain typical of the disintegrins present in viper venoms and instead resembles the CysR of ADAMs.^50^ The smallest recombinant fragment with detectable proteoglycanase activity consists of the Mp/Dis domains,^20,22^ suggesting that these two domains compose a structural as well as functional unit. Mutagenesis studies followed by functional assays using truncated versican as a substrate identified two adjacent lysine residues (^532^KK^533^) in ADAMTS5 as an exosite (Figure 5A).^51^ It is not known if the homologous sequence in ADAMTS4 (^485^KH^486^) also represents an exosite.

As mentioned above, the structures of the domains C-terminal to the Dis have not been reported, but their fold has been predicted with reasonable confidence by AlphaFold (AF-O75173-F1 and AF-Q9UNA0-F1) (Figure 5A).

The CysR (residues 576–685 in ADAMTS4 and 623–731 in ADAMTS5) contains 10 cysteine residues (Figure 5B). According to the AlphaFold model (ID: AF-Q9UNA0-F1), in ADAMTS5 the CysR contains three antiparallel β-sheets and one α-helix (Figure 5C), an arrangement which seems to be preserved in ADAMTS4. The solved crystal structure of ADAMTS13 CysR (PDB 6QIG), on the other hand, is quite divergent (Figure 5D). Since deletion of the CysR severely reduced both aggrecanase^20^ and versicanase activity,^22^ this domain is likely to be involved in substrate recognition, as shown for ADAMTS13,^48^ but no specific exosites have been identified so far. In ADAMTS13, the CysR contains a small hydrophobic exosite (^472^AAV^474^) (Figure 5D)^52^ which is not conserved between the two aggrecanases, being replaced by more hydrophilic residues (Figure 5B).

The Sp (residues 686–837 and 732–874 in ADAMTS4 and ADAMTS5, respectively) is essentially cysteine-free and consists of 10 β-strands in a jelly-roll topology (Figure 5E). While residues in the beta-strands are conserved between ADAMTS4 and ADAMTS5, those in the interconnecting loops are not,^22^ as shown by a superimposition of the Sp domains of ADAMTS4 and -5 (as predicted by AlphaFold) with those of ADAMTS13 (resolved by X-rays) (Uniprot ID: 3GHM and 6QIG) (Figure 5F).^48,53^ This suggests that the overall fold of the Sp domain is conserved among the three family members, whereas the exposed loops contain substrate-specific exosites which can be exploited for selective inhibition. That this is indeed the case was demonstrated when loops β1-β2, β9-β10, and β3-β4 in ADAMTS4 and ADAMTS5 were swapped with those of ADAMTS13, which is unable to cleave proteoglycans.^22^ Two of the resulting chimeras showed a severe reduction in versicanase activity: the exosites comprised residues 717–724 and 788–795 in ADAMTS4 (loops β3-β4 and β9-β10) and 739–744 and 837–844 in ADAMTS5 (loops β1-β2 and β9-β10). Importantly, these exosites were involved in cleavage of both versican and aggrecan (at least in the case of ADAMTS5), suggesting similarities in substrate recognition between these two proteoglycans. From these studies we can conclude that a general feature of aggrecanase exosites is a preference for hydrophilic, positively charged residues (Table 1).

**Table 1:** Exosites in ADAMTS4 and ADAMTS5^a^

enzyme	region	exosite	ref
**ADAMTS4**	Sp	^717^QGNPGHRS^724^	(22)
**ADAMTS4**	Sp	^788^AGNPQDTR^795^	(22)
**ADAMTS5**	Sp	^739^NKKSKG^744^	(22)
**ADAMTS5**	Sp	^837^TDPTKPLD^844^	(22)
**ADAMTS5**	Dis	^532^KK^533^	(51)

a Abbreviations: Dis, disintegrin-like domain; Sp, spacer domain.

Overall, the structural and functional data summarized in this section highlight the presence of distinct differences between ADAMTS4 and ADAMTS5, in particular in exosite preferences (Table 1), as well as between aggrecanases and other metalloproteinases in clan MA such as MMPs and ADAMs, that can be leveraged to achieve highly selective aggrecanase inhibitors.

### 1.3 Targeting Aggrecanases: Zinc Chelation versus Exosite Inhibition

As described in the previous section, the geometry of the active site, in particular that of the histidine triad coordinating the catalytic zinc, is widely conserved in metalloproteinase clan MA, while the enzyme subsites represent specificity determinants among the different members of this superfamily. Accordingly, the selectivity of an active-site inhibitor is determined by its ability to establish interactions with the enzyme subsites. If the affinity for the zinc ion is the driving force in the binding energy between enzyme and inhibitor, as is the case for hydroxamate- and carboxylate-based inhibitors, finely tuning selectivity is a daunting task.

Exosite inhibitors offer a solution to the selectivity issue by targeting highly divergent sequences. Small-molecule exosite inhibitors may suffer from their limited contact area (on average 1000 Å^2^)^55^ and therefore may show limited affinity/inhibitory potency for their target protease if the exosite is relatively extended. As a comparison, complexes between ligands and exosites in thrombin span from 300 to 1700 Å^2^.^56^ Macromolecular inhibitors are characterized by much larger contact areas (1500–3000 Å^2^)^55^ and therefore are ideally suited to target exosites. Not all non-zinc chelating inhibitors are exosite inhibitors (since they may target subsites in the aggrecanase Mp domain), but all exosite inhibitors act via a non-zinc binding mechanism (since they target substrate-binding residues in the ancillary domains).

Compared to other protease families,^56^ identification of exosites in the ADAMTS family is still at its infancy. So far, only in the case of ADAMTS13, have the ancillary domains been structurally resolved.^48,53^ From a practical point of view, this means that rational designing of exosite inhibitors for aggrecanases have been virtually non-existent; instead, exosite inhibitors have been identified by structure–activity relationship (SAR)^51^ or by relying on alternative technology platforms, such as phage display,^27^ that are able to probe the 3D landscape of the target enzyme by screening large libraries of molecules. *De novo* protein structure prediction with AlphaFold^54^ can inform the design/*in silico* screening of exosite inhibitors if the exosite sequences are functionally validated, for example with a quantitative substrate cleavage assay. Assays employing native or full-length substrates are ideally suited to identify exosite inhibitors, which may not be identified when short peptide substrates are used; at the same time, such assays more closely reflect the inhibitory potency of the molecule under physiological conditions, although an important caveat here is that it is very difficult to estimate physiological protein concentrations, in particular for ECM substrates such as proteoglycans. Remarkably, the distinction presented here between active-site inhibitors versus exosite inhibitors supersedes the classical classification into competitive versus non-competitive inhibitors which is substrate-dependent (i.e., the mechanism of inhibition may be different if a either a peptide or protein substrate is used in the assay).

## 2 Zinc-Chelating Inhibitors

The high structural homology among the Mp domains of clan MA metalloproteinases is one of the factors that have hampered the development of selective aggrecanase inhibitors. Nevertheless, as discussed in the previous section, some specific structural features such as the shape of S1′ specificity pocket or the conformation of S2′ loop, offer some opportunity for the design of small molecules with a biased if not selective inhibitory profile.

The classical approach to design metzincin inhibitors relied on the use of zinc metal chelating groups such as hydroxamates and carboxylates. As a result, inhibitors with activity in the nanomolar and picomolar ranges have been identified. Unfortunately, often these molecules were broad-spectrum inhibitors, active also against MMPs and ADAMs, and responsible for off-target toxicity.

Zinc-chelating inhibitors of aggrecanases can be classified as either small molecules or endogenous protein inhibitors such as TIMP3 (Figure 1).

### 2.1 Small-Molecule Inhibitors

The first aggrecanase inhibitors were inspired by the classical structure of metzincin inhibitors, constituted by an aromatic backbone, able to interact with the S1′ and/or S2′ pockets of the enzyme, and a zinc-binding group (ZBG) able to coordinate the catalytic zinc ion. The most used ZBG is the hydroxamic acid. The high affinity for the catalytic zinc (up to picomolar) combined with the conserved geometry of the active site in clan MA of metalloproteinases, often results in a poor selectivity of zinc-binding inhibitors. For example, GM6001 (Ilomastat) is also a potent inhibitor of neprilysin, leucine aminopeptidase, and dipeptidylpeptidase III, three metalloproteases distantly related to its target MMPs.^57^ Here, we classify the small-molecule inhibitors of aggrecanases on the basis of their ZBGs into hydroxamate inhibitors, carboxylate inhibitors, hydantoins, inhibitors with sulfur-based ZBGs, and inhibitors with non-canonical scaffolds.

#### 2.1.1 Hydroxamate Inhibitors

**Figure 6:**
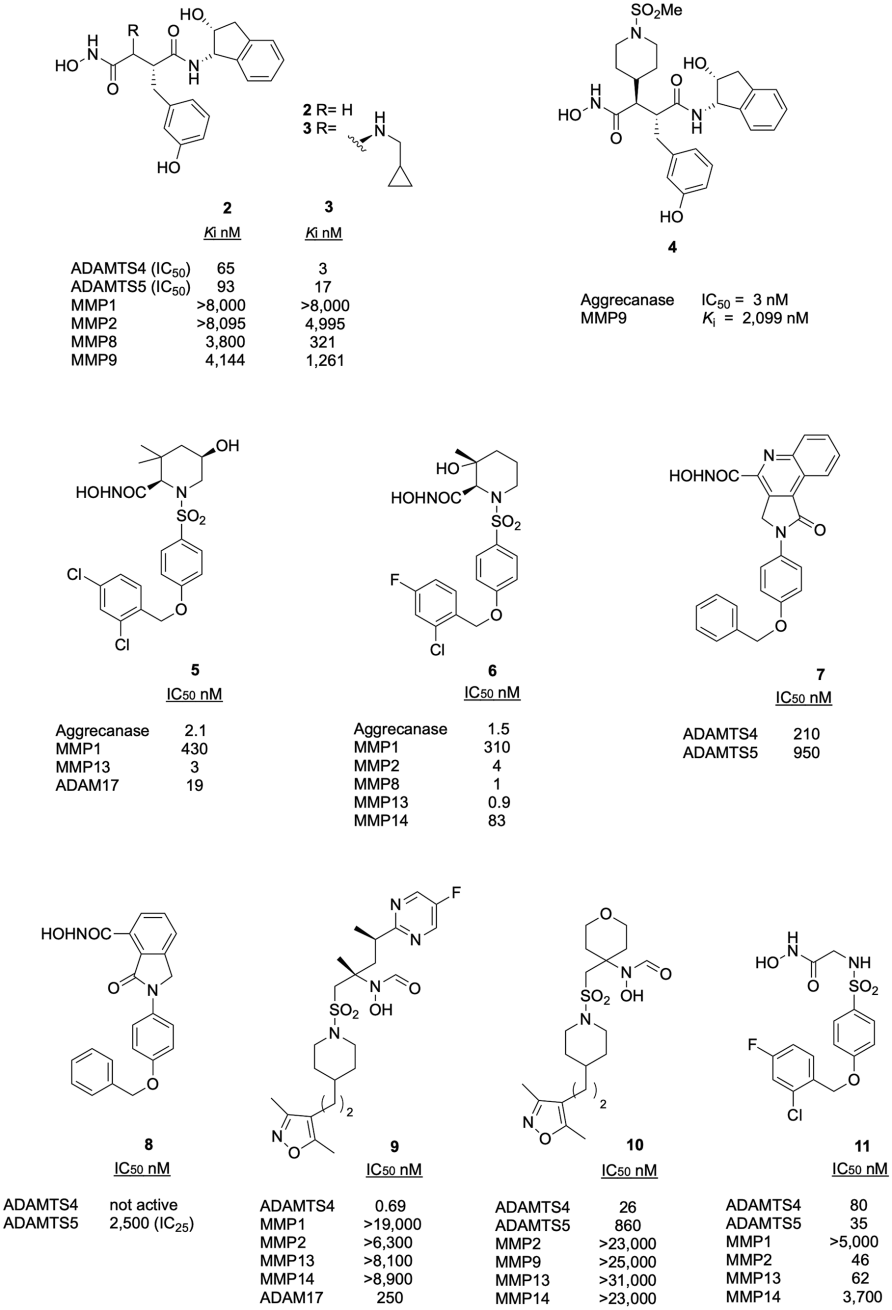
Inhibitory activity and selectivity profile of hydroxamate inhibitors of aggrecanases. IC_25_ indicates the inhibitor concentration achieving 25% activity.

For several years, the absence of structural information about ADAMTS4 and ADAMTS5 together with the lack of suitable screening assays have hampered the design of selective aggrecanase inhibitors. The first molecules tested against aggrecanases were hydroxamate-based MMP inhibitors. In 2001, Yao et al. identified hydroxamate **2** (Figure 6) as an inhibitor of partially purified aggrecanase activity using a structure-based approach.^58^ The succinate-derived peptidomimetic structure of **2** was inspired by substrate specificity of MMP8 which is endowed with limited aggrecanolytic activity.^59^ The introduction of a Tyr residue in P1′ position of the peptide hydroxamate scaffold and the shift of the pseudotyrosine hydroxyl group from *para* to *meta* position improved the inhibitor potency as well as selectivity over MMPs. Moreover, in P2′ position a rigid structure was introduced in compound **3**, resulting in increased potency and selectivity over MMP8. Minor modifications of the P1 side chain also affected selectivity. Compounds **2** and **3** showed good inhibitory potency against isolated ADAMTS4 and -5; in particular, compound **3** displayed lower IC_50_ values than **2** (Figure 6).^60^

**Figure 7:**
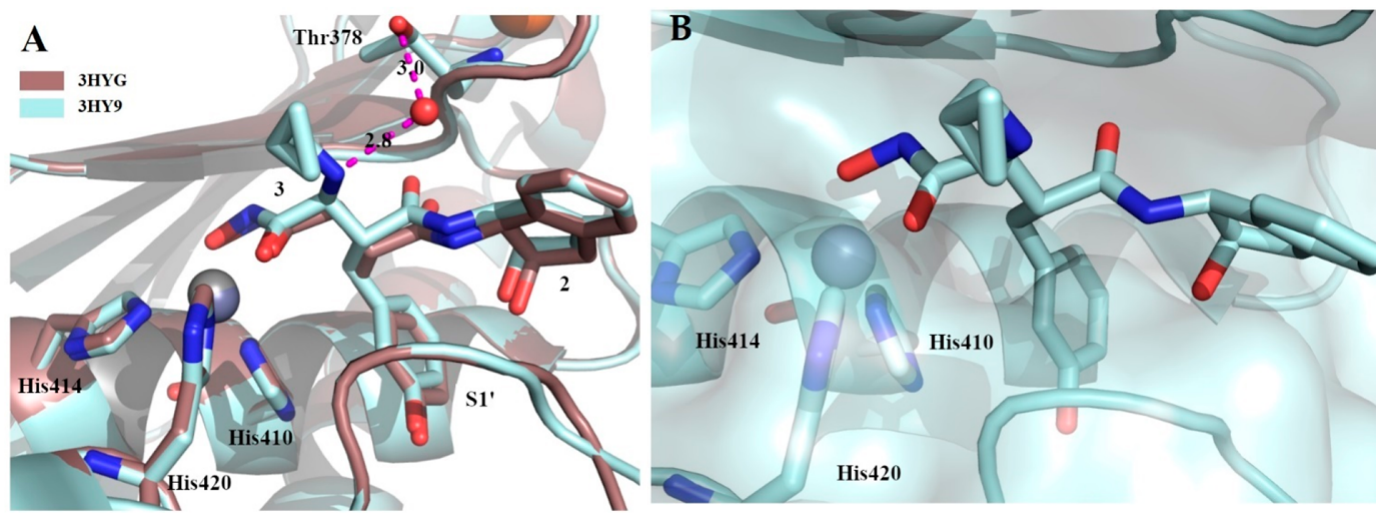
Complexes of compounds **2** (PDB 3HYG) and **3** (PDB 3HY9) with the ADAMTS5 Mp domain. (A) Superimposition between the crystal structures of compounds **2** and **3**; hydrogen bonds are highlighted by pink dashes. (B) Zoom of ligand **3** bound to active site. The zinc ion is shown in gray.

Crystal structures of compounds **2** and **3** in complex with the ADAMTS5 Mp domain showed that the ligands bound to the active site in a similar manner.^60^ The hydroxamate group coordinated the catalytic Zn^2+^ in a standard geometry, thus orienting the phenolic ring into the small S1′ pocket and locating the 2-indanol ring in a specific position further stabilized by several hydrogen bonds (Figure 7). This conformation may justify the selectivity profile of compounds **2** and **3** (Figure 6). The higher inhibitory potency of inhibitor **3** could be explained by an additional hydrogen bond between the −NH group of the cyclopropyl-*N*-methyl methanamine chain and a water molecule connected to Thr378 (Figure 7).

Since replacing the aromatic ring with a biphenyl moiety in P1 did not result in any improvement in activity and selectivity profiles,^61^ Cherney et al. inserted cyclic P1 groups, identifying the *N*-methansulfonyl piperidine **4** (Figure 6) as the most potent aggrecanase inhibitor of the series with selectivity over MMP9.^62^

The first sulfonamido-based aggrecanase inhibitors containing a pipecolic scaffold were reported by Noe et al. in 2005 in two papers exploring different series of hydroxamate-based inhibitors: the 3,3-dimethyl-5-hydroxypipecolic and the 3-OH-3-methylpipecolic series (Figure 6).^63,64^ Dimethyl-5-hydroxypipecolic inhibitors, selective for aggrecanases and the collagenase MMP13, were inspired by a screening on previously published ADAM17 inhibitors. The best inhibitor was compound **5** (Figure 6), for its excellent inhibitory activity on aggrecanases and MMP13, sparing MMP1.^63^ In the 3-OH-3-methylpipecolic series, the best inhibitor was **6** (Figure 6) presenting a 2-chloro-4-fluorobenzyloxyphenyl function in P1′ with good inhibitory activity for the aggrecanases and MMP13, but poorly selective over MMPs.^64^

The exploration of different structures by Cappelli et al. in 2010 led to the design, synthesis, and biological evaluation of a small series of aggrecanase inhibitors, based on a central planar scaffold containing oxoisoindoline or pyrrolo[3,4-*c*]quinolin-1-one, bearing a 4-(benzyloxy)phenyl substituent and different ZBGs.^65^ Derivatives **7** and **8** (Figure 6) exhibited the highest activity against the two aggrecanases. Interestingly, the simplified structure of oxoisoindoline derivative **8** lacked inhibitory activity against ADAMTS4, while maintaining micromolar activity for ADAMTS5. Unfortunately, no selectivity profile over MMPs/ADAMs was reported for this series.

A series of *N*-hydroxyformamide inhibitors was investigated as ADAMTS4 inhibitors.^66^ Starting from a screening of previously published MMP13 inhibitors, the *N*-hydroxyformamide group was identified as a key structural element for ADAMTS4 inhibition. This led to the synthesis of two series of compounds, functionalized by either a phenylpiperazine or a benzyloxypiperidine group. The best compound was the dimethylisoxazolyl derivative **9** (Figure 6), displaying picomolar activity for ADAMTS4 and good selectivity over MMPs. No selectivity data for ADAMTS5 were reported. Compound **9** was crystallized in complex with the Mp domain of ADAMTS1, here chosen as a proxy for ADAMTS4. By combining the results from the crystallographic analysis with a homology model of the ADAMTS4 active site, the *ortho*-methyl substituent on the aromatic ring of P1′ was identified as a crucial moiety for ADAMTS4 inhibition. Later, the P1′ group of **9** was further modified to improve its bioavailability.^67^ The best compound of this series was **10**, being selective for ADAMTS4 over ADAMTS5/MMPs and showing good pharmacokinetic properties as well as *in vivo* efficacy in a spontaneous OA model. In 2013, the arylsulfonamido-hydroxamate **11** (Figure 6) was identified as an inhibitor of aggrecanases and MMP13, with high selectivity over other MMPs.^68^ The inhibitory activity against ADAMTS5, initially tested using a quenched fluorescent (QF) peptide substrate, was further confirmed using purified aggrecan. Inhibition of aggrecan cleavage was significantly decreased (∼2-fold) compared with that of the peptide substrate, a phenomenon frequently observed with small-molecule inhibitors. Compound **11** was able to inhibit aggrecan breakdown in porcine cartilage explants stimulated with interleukin (IL)-1α with almost complete inhibition observed at 10 μM, and with no toxicity effects.^68^

#### 2.1.2 Carboxylate Inhibitors

**Figure 8:**
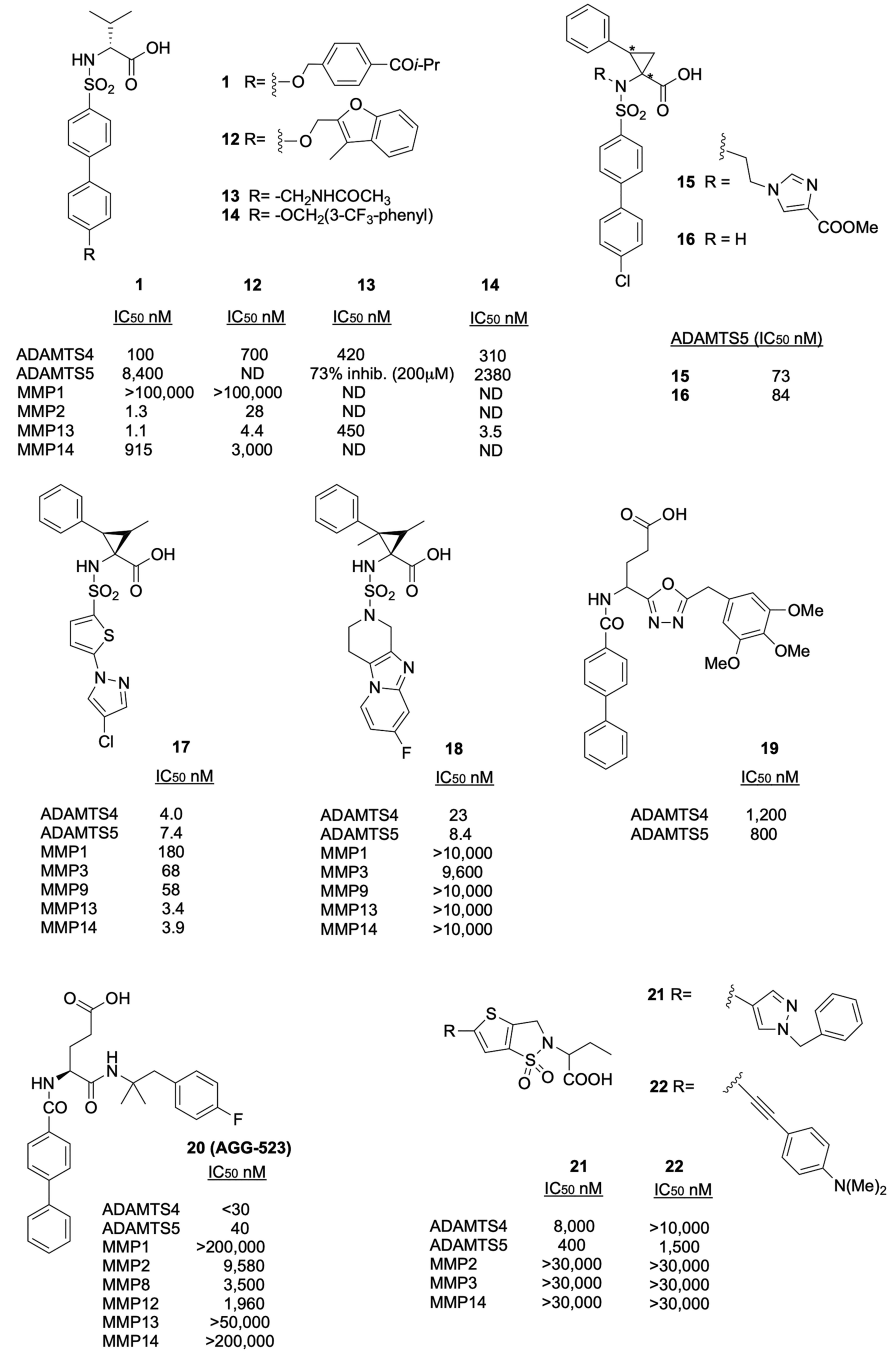
Inhibitory activity and selectivity profile of carboxylate inhibitors of aggrecanases. ND, not determined.

The carboxylate is a viable option as a ZBG since its lower affinity for Zn^2+^ compared to the hydroxamate provides more opportunities for selectivity,^69^ given that the binding energy of the interaction with its target protease will be more evenly distributed between the ZBG and the P substituents. In 2006, researchers at Wyeth reported the first aggrecanase inhibitors bearing a carboxylic acid as a ZBG.^70^ This series presented a biphenylsulfonamido-3-methylbutanoic acid scaffold and was designed on the basis of high-throughput screening (HTS) results and a homology model of ADAMTS4 Mp domain derived from the structure of metalloprotease Atrolysin C. The broad-spectrum MMP inhibitor CGS27023A (Novartis) was docked into the ADAMTS4 active site. In the following SAR analysis, carboxylate **12** (Figure 8) was identified as the best ADAMTS4 inhibitor, sparing MMP1 and MMP14, but still inhibiting MMP2 and MMP13. No data were reported for ADAMTS5, although the parental compound CGS27023A was inactive against this aggrecanase at concentrations up to 25 μM. Compound **12** showed promising pharmacokinetics properties, with a good oral bioavailability and dose–response inhibition of aggrecan degradation in bovine IL-1α- stimulated cartilage explants.

Investigation on the SAR of a variety of substituted aromatic systems, particularly on the para position of the biphenyl ring of biphenyl-4-sulfonamido carboxylates, identified the 4-isobutyryl derivative **1** (Figure 2A and Figure 8) as a nanomolar inhibitor of ADAMTS4 and MMP13, with good selectivity over ADAMTS5, MMP14, and MMP1.^71^

Starting from the structure of **1**, different substitutions to replace the second ring of the biphenyl moiety were investigated.^72^ As a first step, heterocycles were employed such as pyridine, furan, and tetrazole, but the result was a loss of activity against ADAMTS4. Functional groups containing hydrogen bond acceptors and donors were subsequently inserted in the *meta* and *para* positions of the biphenyl ring. The acetamido derivative **13** (Figure 8) was identified as a potent inhibitor of ADAMTS4. The last modification was the insertion of a substituted benzyloxy functionality. The best compound was the trifluoromethyl derivative **14** (Figure 8) which displayed nanomolar activity against ADAMTS4 and MMP13 and 87% inhibition of aggrecan degradation at 10 μg/mL.

In the years from 2009 to 2011, sulfonamido-based cyclopropane carboxylates were investigated as ADAMTS5 inhibitors. These compounds were characterized by a specific P1′ group with novel piperidine or piperazine-based heterocycles connected to a cyclopropane amino acid scaffold via a sulfonamide linkage. The first series of *N*-substituted 2-phenyl-1-sulfonylaminocyclopropane carboxylates was reported with the specific enantiomeric configuration *1R,2S*. The best compound of this series was **15** (Figure 8) with an IC_50_ value of 73 nM against ADAMTS5.^73^ A SAR of non-*N*-substituted 2-phenyl-1-sulfonylaminocyclopropane carboxylates identified compound **16** (Figure 8) with an IC_50_ value of 84 nM against ADAMTS5. In sharp contrast to the previous series, the preferred cyclopropane configuration for the ADAMTS5 activity of compound **16**, and in general of the non-*N*-substituted series, was *1S,2R*. The key points for stereochemical activity were the different orientation of the sulfonamide nitrogen toward the solvent (compound **15**) or a hydrogen bond to the backbone carbonyl of Gly380 residue in the absence of *N*-substitution (compound **16**). A further hit optimization based on the structure of compound **16** was undertaken by modification of the arylsulfonyl moiety and the cyclopropane core. The best compound was **17** (Figure 8), which presented a chloro-imidazole phenyl ring on the sulfonyl group and a *cis*-3-methyl substitution on the cyclopropane. Compound **17** was a potent inhibitor of both ADAMTS4 and ADAMTS5, but, notwithstanding a good selectivity over MMP1 and ADAM17, was equally potent against MMP13 and MMP14. In order to improve the selectivity of **17**, the authors explored the effects of different substituents on thiophene and pyrazole rings and then replaced them with a condensed tricyclic scaffold.^74^ The most promising compound, **18**, contained a methyl group at the 2-position of the cyclopropane ring and a novel P1′ heterotricycle sulfamide-based scaffold (1,2,3,4-tetrahydropyrido-(3′,4′:4,5)imidazo[1,2-*a*]pyridine). Carboxylate **18** showed IC_50_ values of 23 and 8.4 nM against ADAMTS4 and ADAMTS5, respectively, and an improved selectivity over other MMPs (>1000-fold). Docking of **18** into ADAMTS5 and MMP14 Mp domains provided an explanation for this remarkable selectivity. While the cyclopropane ring interacted favorably with Thr378 of ADAMTS5, the 2-methyl substituent provided steric repulsion with Phe198 of MMP14.

Following the design of the P1′ substituted bicyclic ring, Peng et al. reported a series of 4-(benzamido)-4-(1,3,4-oxadiazol-2-yl)butanoic acids as aggrecanase inhibitors.^75^ In this series, a highly rigid 1,3,4-oxadiazol-2-yl ring was introduced as a linker between the scaffold (composed by the carboxylic acid ZBG and the biphenyl P1′ group) and the aromatic P2′ group. The best compound was the biphenyl derivative **19** (Figure 8) with a trimethoxy phenyl moiety as a P2′ interacting group and inhibitory activity in the low micromolar range against ADAMTS4 and ADAMTS5. No selectivity data for MMPs were reported.

Another glutamate-like compound, **20** (AGG-523, US Patent WO2007008994) (Figure 8), developed by structure-based drug design by Wyeth (now Pfizer) and moderately selective for ADAMTS4 and ADAMTS5 over MMPs, is so far one of the few aggrecanase inhibitors reaching clinical trials. Notwithstanding its protective effect in a rat model of surgery-induced OA,^76^ development of AGG-523 was halted following phase I clinical trials in patients with mild to moderate (Clinical Trials ID: NCT00427687) and severe (NCT00454298) knee OA. The two studies were completed in 2008, but no results were reported. Sadly, the inconsistency between the performance of aggrecanase inhibitors in *in vivo* models and clinical trials is a common setback in the pharmaceutical field and highlights once again the need for improved preclinical models and a better understanding on the pathogenesis of OA (see section 4).

An alternative scaffold containing a central thienosultam (1,1-dioxothieno[2,3-*d*]isothiazole) was reported by Atobe et al.^77^ These compounds presented different aromatic, polyaromatic, biphenyl, and alkyne substituents in P1′. The best inhibitors were the *N*-benzyl derivative **21** and the alkyne derivative **22** (Figure 8), which showed good selectivity for ADAMTS5 over ADAMTS4 and MMPs. The best oral bioavailability in rats was reported for carboxylate **21**.

#### 2.1.3 Hydantoin Inhibitors

In order to improve both the selectivity and the pharmacokinetic profile of aggrecanase inhibitors, novel ZBGs alternative to classical hydroxamate and carboxylate were explored.

**Figure 9:**
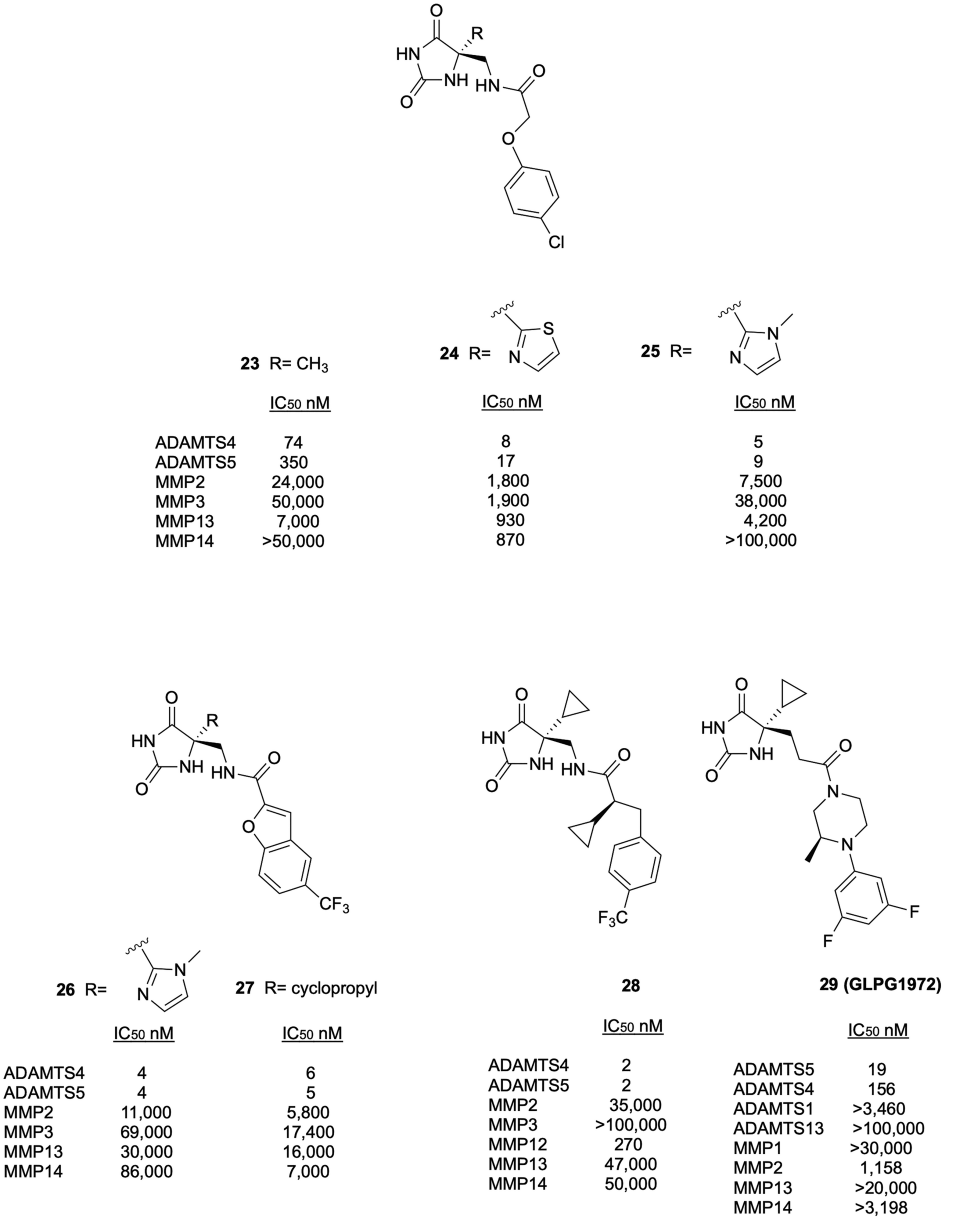
Inhibitory activity and selectivity profile of hydantoin-based aggrecanase inhibitors.

**Figure 10:**
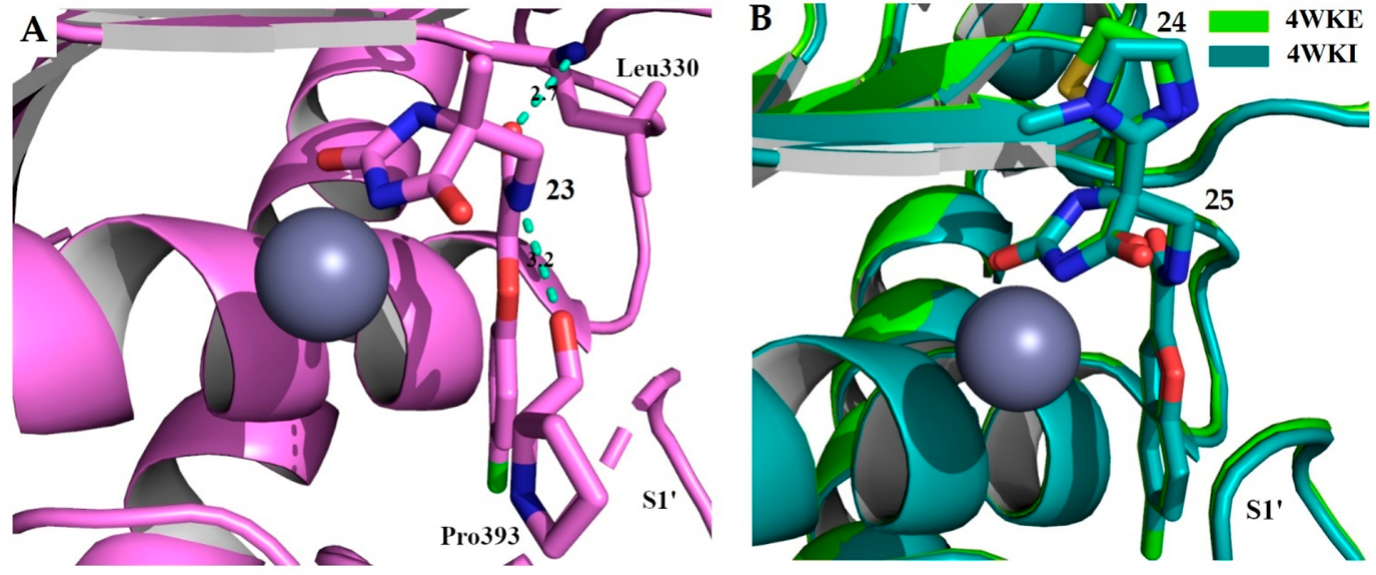
Complex of compounds **23** (PDB 4WK7), **24** (PDB 4WKE), and **25** (PDB 4WKI) with the ADAMTS4Mp domain. (A) Zoom of compound **23** into the active site; hydrogen bonds are highlighted by green dashes. (B) Superimposition between compounds **24** and **25** bound to ADAMTS4 Mp domain. The zinc ion is shown in gray.

After HTS of more than 80 000 structurally different compounds, researchers at Eli Lilly identified hydantoin **23** (Figure 9) as an alternative ZBG to develop aggrecanase inhibitors.^78^ The X-ray structure of compound **23** in complex with ADAMTS4 showed that the hydantoin ring coordinates the Zn^2+^ ion while the amide linker established hydrogen bonds with Leu330 and Pro393, thus orienting the aromatic ring into the S1′ pocket (Figure 10A). This crystallographic analysis provided fundamental information to address the P1 substitution using structure-based drug design to improve selectivity. Modifying P1 from methyl (compound **23**) to thiazole or imidazole group (compounds **24** and **25**, respectively, Figure 9) resulted in increased selectivity for ADAMTS4 and ADAMTS5 over MMPs. The crystal structures of thiazole (**24**) and imidazole (**25**) derivatives in complex with ADAMTS4 showed that they bound to the active site in a similar manner, a slight difference being detectable only around the imidazole ring that was rotated 45° out of the plane occupied by the thiazole (Figure 10B).

Benzofuran derivative **26** (Figure 9) was identified as the best inhibitor of this series, with an IC_50_ value of 4 nM for ADAMTS4 and ADAMTS5 and good efficacy in a rat model of inflammatory OA.

On the basis of these results, Eli Lilly’s researchers further optimized the benzofuran hydantoin scaffold by introducing a cyclopropyl substituent in P1′ position to obtain compound **27** (Figure 9).^79,80^ Compound **27** revealed a good projected human pharmacokinetic profile but a significant, undesired glutathione conjugation in rats. With the aim of minimizing glutathione conjugation and lowering the projected human dose, the structure of **27** was further modified by replacing the benzofuran moiety. *para*-Trifluoromethyl benzyl derivative **28** (Figure 9) was finally identified as the most promising aggrecanase inhibitor with nanomolar activity against ADAMTS4 and ADAMTS5, good selectivity profile over MMPs, good pharmacokinetic profile, and efficacy in a rat model of inflammatory OA.

**Figure 11:**
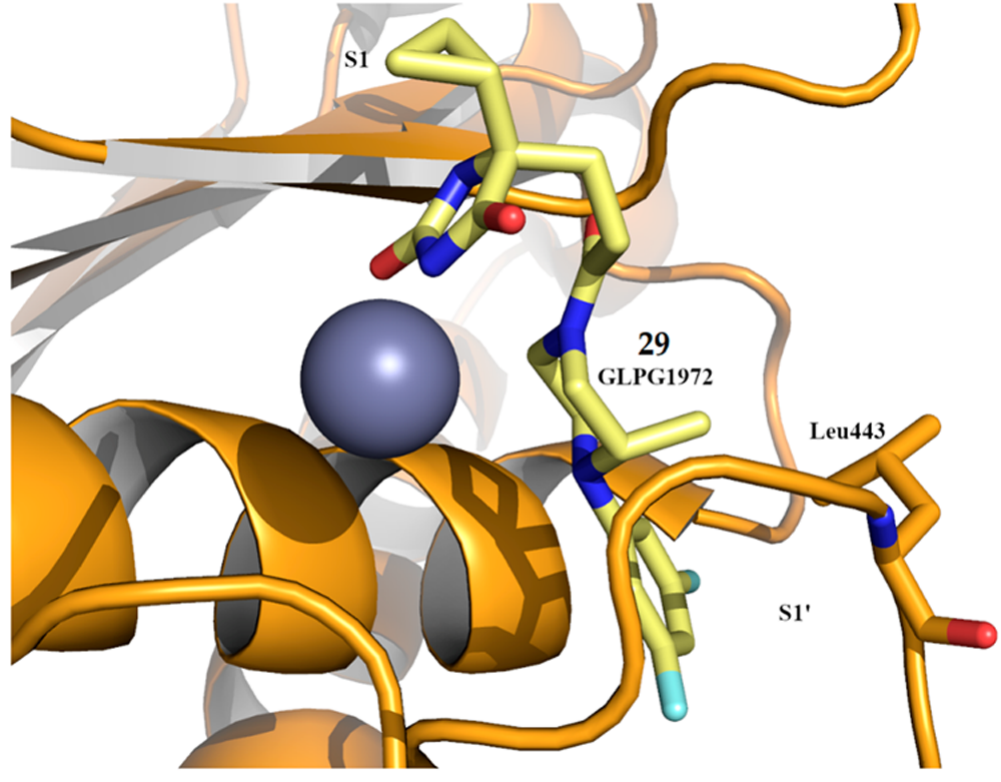
Crystal structure of compound **29** (PDB 6YJM) in complex with the ADAMTS5 Mp domain. The zinc ion is shown in gray.

In 2021, a new hydantoin-based ADAMTS5 inhibitor, GLPG1972/S201086 (**29**, Figure 9), bearing a difluorophenyl-piperazine as P1′ group, was co-developed by Galapagos and Servier.^81^ The crystal structure of **29** in complex with the ADAMTS5 Mp domain showed, in agreement with other similar derivatives, that the hydantoin ring coordinated the Zn^2+^ ion thus orienting the cyclopropyl ring toward the S1 pocket while the difluorophenyl ring perfectly fitted the S1′ pocket. The specific conformation of the methyl group, axial to the piperazine ring, established hydrophobic contacts with Leu443 (Figure 11). GLPG1972 had IC_50_ values of 19 and 156 nM against ADAMTS5 and ADAMT4, respectively, and good selectivity over MMPs and ADAM17. In mouse cartilage explant assays, the IC_50_ value increased 100-fold (10 μM),^81^ most likely reflecting reduced target engagement and/or competition with aggrecan. This reduced efficacy in cartilage explant assays compared with pure component assays has been frequently observed for aggrecanase inhibitors.^82^ No inhibition was observed on type II collagenolysis in both mouse and human cartilage explants or on MMP-mediated aggrecan degradation, thus confirming GLPG1972 selectivity over MMPs.^83^ In a mouse model of surgery-induced OA, GLPG1972 at 30–120 mg/kg reduced femorotibial aggrecan loss, cartilage structural damage, and subchondral bone sclerosis (20–40% compared to vehicle controls). Double-blind, placebo-controlled phase I trials were then conducted in Belgium (NCT02612246), USA (NCT03311009), and Japan. GLPG1972 was safely tolerated in healthy adult men (of both white and Japanese origin) and in male and female participants with OA.^84^ In OA patients, once-daily dosing for 14 days significantly reduced levels of ADAMTS-generated aggrecan cleavage (ARGS) fragments in plasma compared with placebo in healthy volunteers. Once GLPG1972 administration was stopped, ARGS levels returned to baseline within 14 days, remaining stable until day 50, suggesting that the interaction between GLPG1972 and ADAMTS5 was reversible. In the light of these promising results, GLPG1972 was evaluated in 932 patients with symptomatic knee OA in a double-blind placebo-controlled randomized phase II clinical trial (NCT03595618). GLPG1972 was given orally at 3 different doses (75, 150, and 300 mg), once daily for 52 weeks and was well tolerated, with no increased risk of adverse MSK events compared with placebo. However, GLPG1972 did not meet its primary end point of change from baseline in cartilage thickness of the medial tibiofemoral compartment, as measured by magnetic resonance imaging at week 52. All secondary outcomes, both structural and pain-related, were not met in this trial. The causes of this lack of efficacy are currently unknown, and GLPG1972 did not progress into phase III trials.

#### 2.1.4 Inhibitors with Sulfur-Based ZBGs

**Figure 12:**
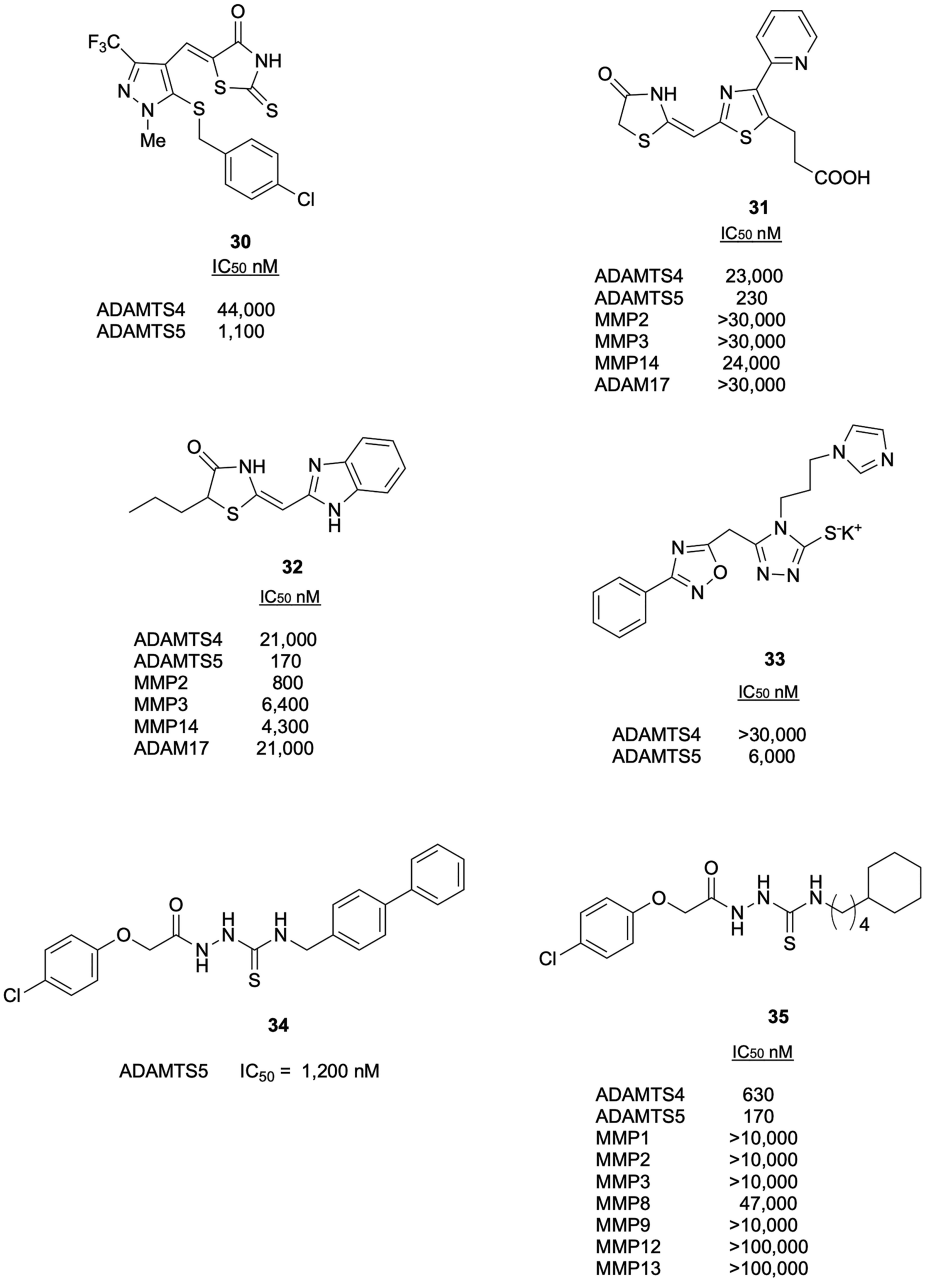
Inhibitory activity and selectivity profile of aggrecanase inhibitors with sulfur-based ZBGs.

An alternative and less explored ZBG is the thioxothiazolidinone, investigated in a class of rhodanine-based ADAMTS5 inhibitors developed by Wyeth.^85,86^ The 5-((3-(trifluoromethyl)-1*H*-pyrazol-4-yl)methylene)-2-thioxothiazolidin-4-one derivative **30** (Figure 12) was identified as the best compound of these series with inhibitory activity in the micromolar range for ADAMTS5 and a modest selectivity over ADAMTS4.^86^ No selectivity profile over MMPs/ADAMs was reported for this series.

Another series of derivatives bearing a thiazolidin-4-one emerged via HTS and led to the identification of the pyridine derivative **31** (Figure 12) as a promising ADAMTS5 inhibitor with a good selectivity profile over ADAMTS4, ADAM17, and MMPs.^87^ Compound **31** was described as a non-competitive inhibitor in a QF-peptide cleavage assay following Lineweaver–Burk plot analysis.^87^ However, given the magnification of experimental errors associated with linear plots compared to nonlinear fitting of untransformed data to the Michaelis–Menten equation,^88^ and the lack of additional functional/structural characterization, it is premature to define compound **31** as a true non-zinc chelating inhibitor. Compound **31** inhibited aggrecan degradation in IL-1-stimulated bovine cartilage explants (IC_50_ value: 22 μM). Unfortunately, **31** exhibited low membrane permeability evaluated by flux through MDCK cells in transwell culture. To address this issue, the structure of compound **31** was modified by removing the carboxylic acid alkyl chain, which was considered responsible for the low membrane permeability, while maintaining the thiazolidinone as ZBG.^89^ The 2-pyridyl thiazole central core was then replaced by various heterocyclic systems (monocycle, bicycle, or tricycle) to investigate the effect on ADAMTS5 inhibition. The benzimidazole derivative **32** (Figure 12) showed improved membrane permeability compared to **31**, but this was achieved at a cost of a loss in selectivity over MMPs and ADAM17.

An alternative ZBG is the 1,2,4-triazole-3-thiol scaffold where the exocyclic sulfur atom coordinates the zinc-ion. From a focused library of 500 differently substituted 1,2,4-triazole-3-thiols, the 3-(*N*-imidazolyl)propyl derivative **33** (Figure 12) emerged as the best ADAMTS5 inhibitor, with good selectivity over ADAMTS4.^90^

Based on the inhibitory activity of the synthetic intermediate acylthiosemicarbazide **34** (Figure 12), a library of 920 analogues with this ZBG was designed.^91^ Different modifications of acetylthiosemicarbazide were explored, the SAR analysis and docking study revealing three fundamental interactions of acylthiosemicarbazide and the ADAMTS5 active site. The best inhibitor was **35** (Figure 12), with nanomolar activity against ADAMTS5 and good selectivity profile over a panel of MMPs, probably caused by an optimized interaction between its cyclohexylbutyl group and the S1′ pocket.

#### 2.1.5 Inhibitors with Non-canonical ZBGs

**Figure 13:**
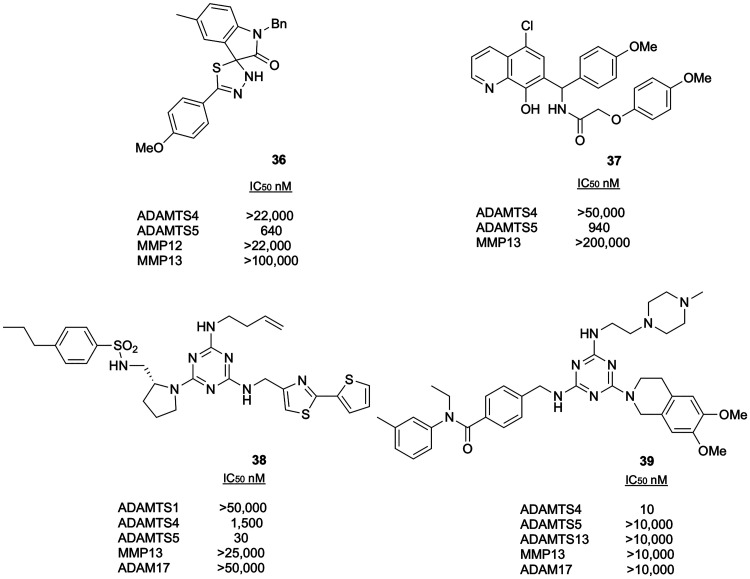
Inhibitory activity and selectivity profile of aggrecanase inhibitors with non-canonical ZBGs.

Following HTS, researchers at Wyeth reported preliminary data on two different series of ADAMTS5 inhibitors using as a scaffold either 5′-phenyl-3′*H*-spiro[indoline-3,2′-[1,3,4]thiadiazol]-2-one^92^ or hydroxyquinoline.^93^ These two series have been investigated through a wide SAR analysis and led to the identification of several ADAMTS5 inhibitors with sub-micromolar potency characterized by a good selectivity over ADAMTS4, MMP12, and MMP13. The best compounds of each series were the spiroindoline **36** and the 8-hydroxychlorochine **37**, respectively (Figure 13).

Researchers at GlaxoSmithKline (GSK) reported the identification from a four-billion-member DNA-encoded 1,3,5-triazine library (Encoded Library Technology) of sulfonamide **38** (Figure 13) as a potent ADAMTS5 inhibitor (IC_50_ value: 30 nM) presenting a >50-fold selectivity over ADAMTS4 and an impressive >1000-fold selectivity over ADAMTS1, MMP13, and ADAM17.^94^ Compound **38** was able to inhibit the release of ARGS aggrecan fragments and GAGs in response to IL-1β/oncostatin M (OSM) stimulation in human OA cartilage explants. No binding/functional experiments were carried out to assess the mechanism of inhibition of **38**. Analysis of the literature allowed El Bakali et al. to define the amino-triazine ring as the ZBG, either via the exocyclic NH group or one of the triazine nitrogen.^95^ The same approach was used to identify a potent, highly selective ADAMTS4 inhibitor, the 3,4-dihydroisoquinoline derivative **39** (Figure 13).^96^

### 2.2 Tissue Inhibitor of Metalloproteinase 3

The proteolytic activity of aggrecanases is regulated by TIMPs. TIMPs act as endogenous, ECM-associated inhibitors of several MA families such as MMPs, ADAMs, and ADAMTSs.^97^ TIMPs are 4 small (21–28 kDa) proteins composed by an inhibitory N-terminal domain of about 125 residues and a C-terminal domain of about 65 residues, each stabilized by 3 disulfide bonds.^98^ The N-terminal domain is a fully active metalloproteinase inhibitor. The mechanism of inhibition involves the insertion of the ridge comprising the N-terminal five residues into the metalloprotease active site in such a way that the first amino acid, which is invariably a cysteine, coordinates the active-site zinc through its α-amino and carbonyl groups.^97^

TIMP3 is the only vertebrate TIMP that is bound to the ECM through electrostatic interactions with sulfated GAGs.^99,100^ TIMP3 expression is post-translationally downregulated in OA.^97^ That a decreased TIMP3 expression may contribute to the proteolytic imbalance typical of the disease was corroborated by the phenotype of *Timp3* null mice, which exhibited mild cartilage degradation in the absence of inflammatory or mechanical insults.^101^ Among the 4 TIMPs, TIMP3 is also the most potent aggrecanase inhibitor (Table 2).^102,103^ A truncated TIMP3 variant containing only the N-terminal domain (N-TIMP3) inhibited the activity of ADAMTS4 and ADAMTS5 against native bovine aggrecan with IC_50_ values of 3.3 and 0.66 nM, respectively.^103^

**Table 2:** IC_50_ Values (nM) for Inhibition of MMPs, ADAMs, and ADAMTSs by Engineered TIMP-3 Variants^a^

inhibitor	MMP1	MMP2	MMP3	ADAM17	ADAMTS4	ADAMTS5	ref
N-TIMP3	1.7	2.7	53.6	13.7	1.8	0.5	(108)^b^
[-1A]N-TIMP-3	800	970	>1000	33.9	22.2	1.7	(108)^b^
TIMP-3	1.2	0.6	1.2	3.54	0.19	1.27	(111)^c^
TIMP-3 K26A/K45A	0.52	0.63	0.92	3.78	0.12	0.95	(111)^c^
TIMP-3 K42A/K110A	0.60	0.60	1.4	2.34	0.24	1.12	(111)^c^
TIMP-3 K22S/F34N	ND	0.9	ND	341	ND	ND	(113)^c^
TIMP-3 H55N/Q57T/	ND	1.0	ND	29	ND	ND	(113)^c^
K71N/E73T/D87N/							
K89T/R115T							
TIMP-3 H55N/Q57T/	ND	2.7	ND	156	ND	ND	(113)^c^
K71N/E73T/D87N/							
K89T/R115T-Fc							
TIMP-3 H55N/Q57T/	ND	1.6	ND	145	ND	ND	(113)^c^
K71N/E73T/D87N/							
K89T/R115T-HSA							
TIMP-3 K26A/K45A-PEG	ND	0.4	ND	123	ND	ND	(113)^c^

a Note that different forms of enzymes were tested in the different studies. Abbreviations: HSA, human serum albumin; ND, not determined.

b Values determined using a recombinant aggrecan fragment comprising the Glu392-Ala393 cleavage site (GST-IGD-FLAG substrate).

c Values determined using a QF-peptide substrate.

Because of its sub-nanomolar affinity, TIMP3 is appealing as a DMOAD. Unfortunately, two factors prevented the use of TIMP3 as a therapeutic agent, i.e., its broad-spectrum inhibitory activity as well as its low half-life. TIMP3 inhibits the majority of MMPs, several ADAMs as well as ADAMTS2.^97,104^ Promiscuous metalloproteinase inhibition has been frequently associated with undesired MSK effects such as arthralgia, myalgia, joint stiffness, and tendinitis.^105^

TIMP3 half-life is negatively regulated by its endocytosis and subsequent lysosomal degradation via the low-density lipoprotein receptor-related protein 1 (LRP1) receptor.^106^ Therefore, it might be desirable to increase TIMP3 half-life to improve efficacy and reduce the dose or frequency of administration under a therapeutic regime. Strategies aiming to engineer TIMP3 as a DMOAD should aim to increase both TIMP3 selectivity and half-life.

Despite its mechanism of inhibition, N-TIMP3 is a more potent inhibitor of ADAMs and ADAMTSs compared with MMPs (Table 2). Introduction of an extra alanine residue at N-terminus of N-TIMP3 further increases this bias by disturbing the interaction between Cys1 and the active-site Zn^2+^ (Table 2).^107^ The resulting variant, [-1A]N-TIMP3, was a nanomolar inhibitor of ADAMTS5, being 13-fold selective over ADAMTS4 and 20-fold selective over ADAM17, while almost sparing MMPs.^108^ [-1A]N-TIMP3 potently inhibited GAG release from knee OA cartilage stimulated with IL1α/OSM (although not as potently as TIMP3), while not affecting MMP-mediated collagen release.^108^ Full-length [1-A]TIMP3 was tested *in vivo* using a model of spontaneous OA, the STR/Ort mice. Transgenic STR/Ort mice over-expressing [1A]TIMP3 either under the elongation factor *EF1α* promoter (ubiquitous expression) or the *Col2a1* promoter (chondrocytes-specific expression) were protected from cartilage degradation compared to wild-type mice at 40 weeks.^109^ In addition, these transgenic mice showed increased trabecular bone mass, suggesting that administration of [1-A]TIMP3 may prevent osteoporotic bone loss, particularly in female mice. [1-A]TIMP3 was also tested in a surgical OA model. Transgenic C57BL/10 mice overexpressing [1-A]TIMP-3 under the *Col2a1* promoter showed increased protection following destabilization of medial meniscus (DMM) as compared to wild-type.^110^ Overexpression of [1-A]TIMP3 was more efficient in protecting from cartilage degradation than that of TIMP3 8 weeks post-DMM, a time point mimicking late-stage OA. Importantly, while mice over-expressing TIMP3 showed a significant decrease in trabecular bone volume, number, and thickness, neither WT mice nor [-1]TIMP3 showed these changes, thus demonstrating that selective inhibition of aggrecanases may prevent unwanted effects on bone integrity.

Recombinant TIMP3 (rTIMP3) had a short half-life (3.6 h) when added to HTB94 chondrosarcoma cells, due to its rapid uptake and degradation by the LRP1 receptor.^106,111^ Based on the notion that LRP1 ligands are characterized by a positively charged cluster composed by two lysine residues 21 Å apart which bind to negatively charged residues on LRP1,^112^ the Troeberg’s group analyzed a panel of TIMP3 variants where pairs of lysine residues predicted to be separated by 21 Å were mutated to alanine to increase TIMP3 half-life.^111^ They identified two variants, TIMP3 K26A/K45A and K42A/K110A, which bound with decreased affinity to LRP1 ectodomain *in vitro* and therefore exhibited an extended half-life when added to HTB94 chondrosarcoma cells.^111^ Importantly, the two variants maintained the inhibitory profile of the parental TIMP3 molecule against several metzincins (Table 2). Most likely due to their resistance to LRP1-mediated endocytosis, TIMP3 variants K26A/K45A and K42A/K110A were more effective than wild type TIMP3 in inhibiting GAG release from porcine cartilage explants following a 3-days pre-incubation period.^111^ Mutations aiming to prevent LRP1 binding exert also positive effects on TIMP3 expression levels,^111,113^ an important factor in view of a future scale-up for industrial production.

If systemic administration of rTIMP3 is attempted, another issue is the short half-life of the molecule in serum. The molecular weight cutoff for glomerular filtration is 30–50 kDa,^114^ well above TIMP3 molecular weight of ∼22 kDa.^97^ Fusion with a human Fc antibody region can extend half-life through the interaction with the immunoglobulin salvage receptor FcRn; the Fc region itself can be engineered to strengthen further this interaction.^115^ A similar effect is produced by fusing with a serum protein with extended half-life such as albumin or by increasing the molecular mass of TIMP3 above the glomerular filtration cut-off, for example by conjugation with polyethylene glycol (PEG) or introduction of additional glycosylation sites. These strategies have been extensively explored by Chintalgattu et al.^113^ A TIMP3 variant (K22S/F34N) containing a mutated lysine to decrease LRP1 affinity together with an additional glycosylation site only showed a modest increase in rat serum half-life compared with wild-type TIMP3 (66 min versus 48 min), while introduction of 5 glycosylation sites (variant H55N/Q57T/K71N/E73T/D87N/K89T/R115T) increased half-life up to 226 min. C-terminal fusion with albumin or Fc dramatically extended the half-life of the 5× glycosylated molecule (720 and 930 min, respectively). Similarly, a PEGylated version of K22S/F34N showed a half-life of 1716 min. These variants have not been tested for their inhibitory activity against aggrecanases (Table 2). This is quite unfortunate since it is likely that extended glycosylation/PEGylation will affect their inhibitory profile. Another approach involved N-terminal fusion of TIMP3 with the latency-associated peptide from the cytokine Transforming growth factor β, which can be removed in situ by MMP1.^116^ The resulting activated TIMP3 molecule has an extra leucine at the N-terminus and, similarly to [-1A]TIMP3, showed higher selectivity for aggrecanases over MMPs, although no inhibition constants have been reported so far (estimated IC_50_ value for ADAMTS4 inhibition from Figure 1E in ref (116) is ∼10 nM, i.e., considerably higher than wild-type TIMP3).

Taken all together, these studies highlighted the feasibility of improving TIMP3 selectivity and pharmacokinetics. The next step will be combining the selectivity profile of [1-A]TIMP3 with the increased half-life of the K26A/K45A and K42A/K110A variants. So far, administration of recombinant TIMP3 has not been tested in mouse models of OA, which have focused on transgenic expression. Therefore, there is an important piece of information missing along the pathway to the therapeutic application of TIMP3 as DMOAD. However, this approach has been investigated in the context of cardiovascular diseases.^117^ For example, to test the protective effect of TIMP3 on myocardial infarction, rTIMP3 has been directly injected into the myocardium of pigs subjected to coronary ligation.^118,119^ In this case rTIMP-3 was administered in a hyaluronan-rich hydrogel, mimicking binding of TIMP3 to GAGs, to extend its half-life.^118^

## 3 Exosite Inhibitors

Avoiding the chelation of the zinc atom, common to all the metalloproteases belonging to clan MA, could be an important factor for improving the selectivity profile and avoiding off-target toxicity.^120,121^ The inhibitors discussed in this section are devoid of a ZBG; some of them have been further characterized as exosite inhibitors.

### 3.1 Sulfated Glycosaminoglycans

**Figure 14:**
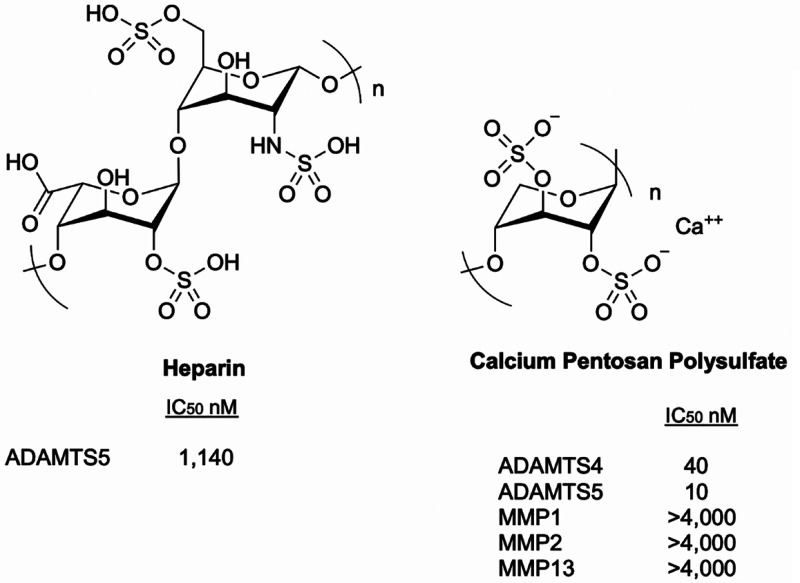
Structure and inhibitory activity of sulfated GAGs as aggrecanase inhibitors.

Sulfated GAGs represent a promising opportunity to achieve exosite inhibition. For example, heparin (Figure 14), a heterogeneous preparation of linear, highly sulfated GAGs, inhibited ADAMTS5 aggrecanase activity with an IC_50_ value of 20 μg/mL.^122^ Since the average molecular weight for porcine heparin is 17.5 kDa,^123^ this translates to an IC_50_ of 1.14 μM. Unfortunately, due to its anticoagulant properties and associated side effects, such as thrombocytopenia,^124^ heparin itself is not suitable as a DMOAD.

An alternative to heparin may be Calcium Pentosan Polysulfate (CaPPS) (Figure 14), a calcium salt form of chemically sulfated molecule produced from beechwood (*Fagus sylvatica*) consisting of a β-1,4-linked polymer of xylose with β 4-methyl glucuronic acid residues attached to the 2-OH of every 10th xylose. CaPPS has been shown to effectively inhibit aggrecan degradation in human OA cartilage explants under inflammatory conditions.^122,125^ CaPPS (molecular weight: 4–6 kDa, average 5.7) inhibited aggrecanase activity of ADAMTS4 and ADAMTS5 with IC_50_ values of 40 and 10 nM, respectively, while sparing MMP1, MMP2, and MMP13 (IC_50_ values >4 μM).^122^ Functional studies using domain-deletion forms of aggrecanases demonstrated that CaPPS binds to the Sp domain of ADAMTS4 and the CysR domain of ADAMTS5.^122^ In cell culture, the mechanism of inhibition of CaPPS is quite complex. By blocking the endocytosis of TIMP3 via the LRP1 receptor, CaPPS increased extracellular TIMP3 levels; it also enhanced the affinity of TIMP3 for ADAMTS4 and ADAMTS5 (>100 fold).^122,125,126^ Although CaPPs has been shown to be effective in some OA clinical trials,^127−129^ it has not been yet approved as a DMOAD. Further clinical trials are under way (NCT04814719, NCT04809376).

### 3.2 Glycoconjugates

GAGs can be successfully linked to canonical metalloproteinase inhibitory scaffolds, such as the arylsufonamide, and ZBGs, thus generating glycoconjugates.^130^

**Figure 15:**
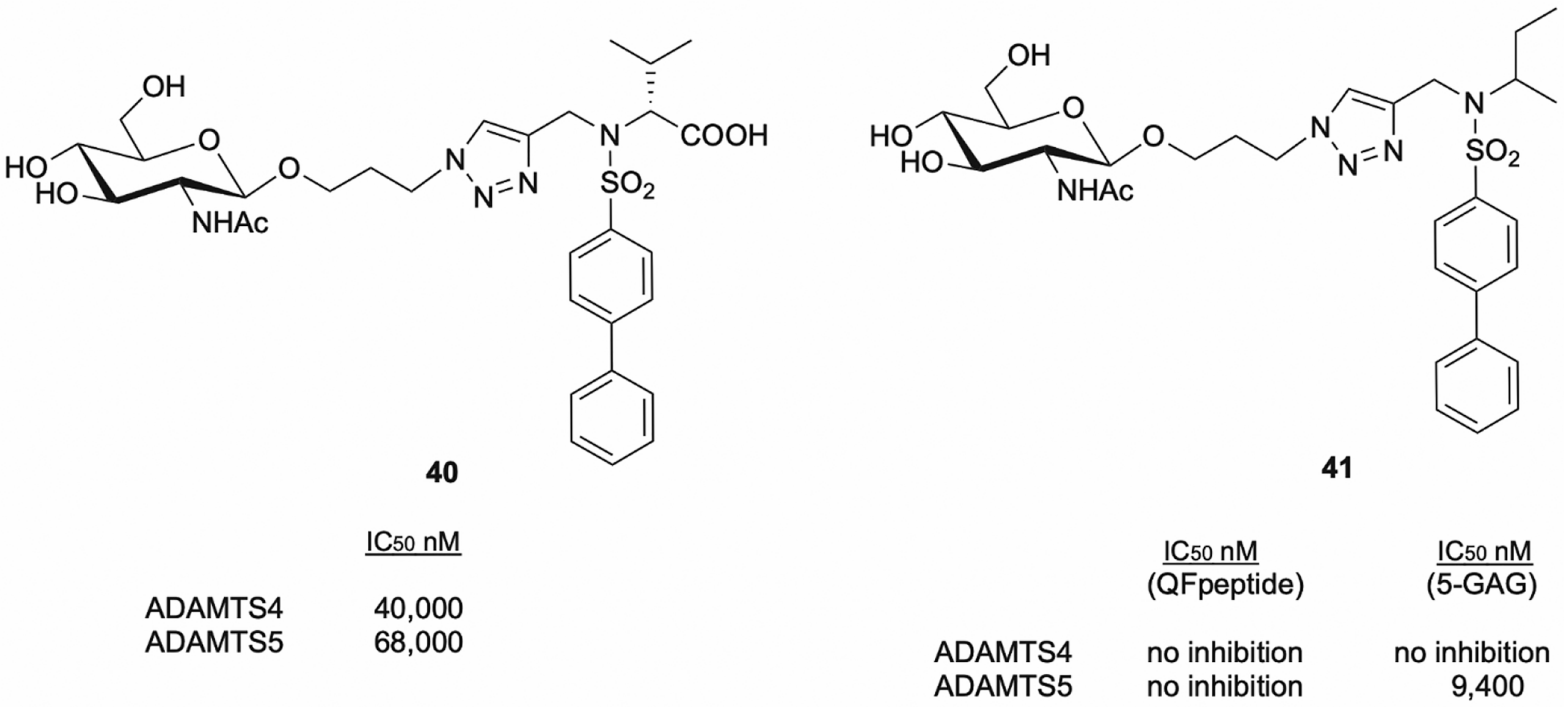
Structure, affinity, and inhibitory profile of glycoconjugates as aggrecanase inhibitors.

By screening a series of glycoconjugate MMP12 inhibitors,^131,132^ Santamaria et al.^51^ identified carboxylic acid **40** (Figure 15), where a β-*N*-acetyl-d-glucosamine monosaccharide is linked to the arylsulfonamide scaffold, as an ADAMTS5 inhibitor with activity in the micromolar range. Removal of the ZBG resulted in compound **41** (Figure 15), which inhibited ADAMTS5 cleavage of both versican and aggrecan with IC_50_ values in the micromolar range, but spared ADAMTS4. No significant inhibition was observed on QF peptide cleavage assays; moreover, **41** enhanced the inhibitory activity of the broad-spectrum zinc-binding MMP inhibitor GM6011 against ADAMTS5. These results suggested the possibility that **41** targets an exosite. Docking calculations combined with molecular dynamics simulations demonstrated that **41** targets the interface of the Mp and Di domains. The combination of kinetic and *in silico* study demonstrated that **41** is an exosite cross-domain inhibitor, acting by an unprecedented mechanism where the S1′ pocket is occupied by the arylsulfonamide scaffold, whereas the sugar moiety interacts with a positively charged cluster (^532^KK^533^) in the ADAMTS5 Dis domain (Figure 5A). Site-directed mutagenesis confirmed that this region represents a previously unknown exosite which is critical for substrate recognition and can therefore be targeted for the development of selective ADAMTS5 inhibitors.

### 3.3 Flavonoids

Several natural compounds such as flavonoids are known to possess metzincin inhibitory activity, and some of them have been investigated as aggrecanase inhibitors.

**Figure 16:**
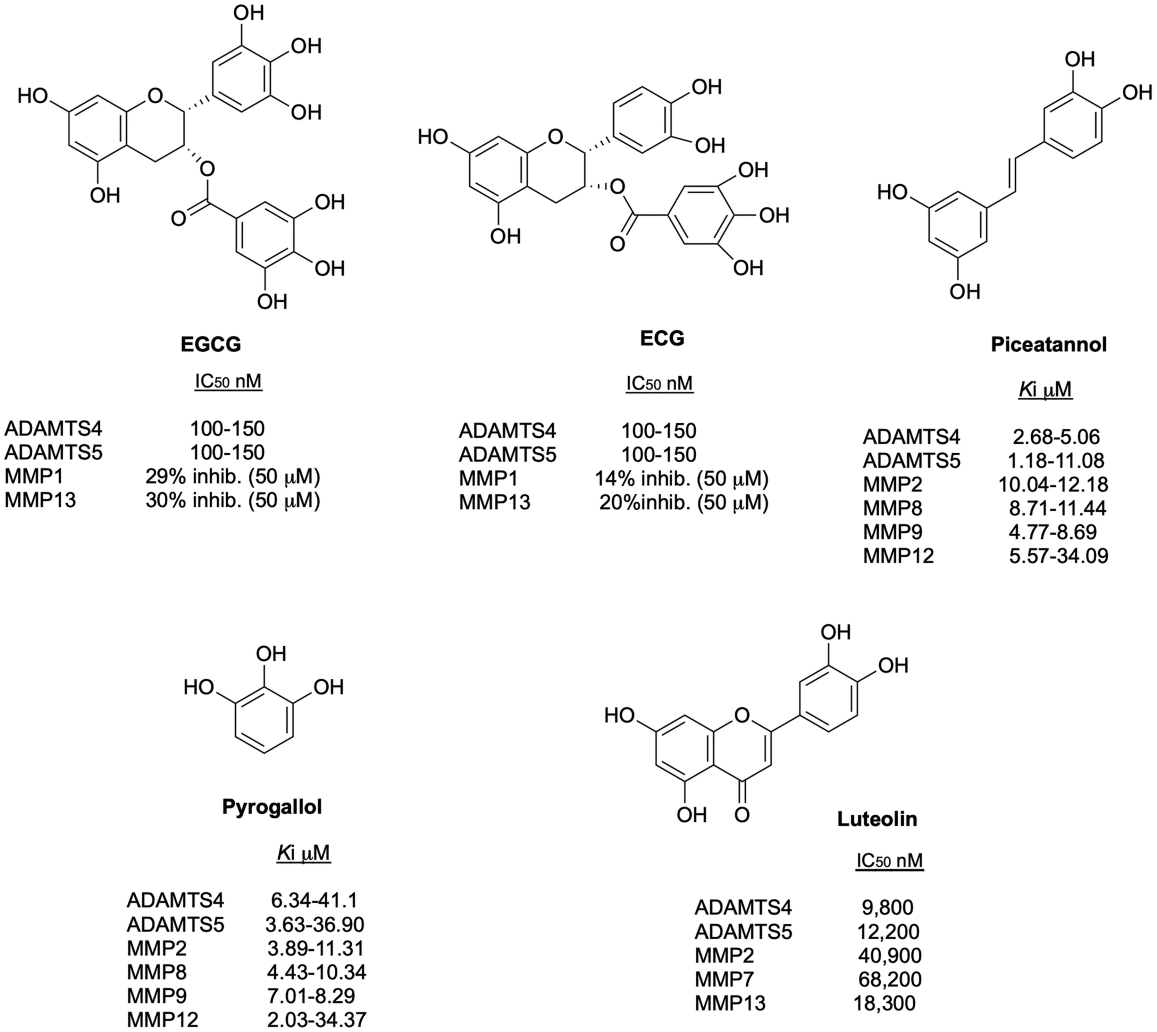
Inhibitory activity and selectivity profile of flavonoid-based aggrecanase inhibitors.

A series of green tea catechin gallate esters have been reported as ADAMTS4, ADAMTS5, and ADAMTS1 inhibitors. In particular, (−)-epigallocatechin-3-gallate (EGCG) and (−)-epicatechin gallate (ECG) and piceatannol (Figure 16) showed IC_50_ values for the two aggrecanases in the range of 100–150 nM, although they were poorly selective over MMPs and ADAMs.^133^ In 2009 Cudic et al.^134^ evaluated molecules that are structural components or structurally related to EGCG, ECG, and piceatannol such as resveratrol, *trans*-stilbene, *cis*-stilbene, deoxyhapontin, rhapontin, pyrocathecol, and pyrogallol (Figure 16). These molecules inhibited aggrecanase cleavage of QF triple-helical peptides with IC_50_ values in the low micromolar range. A poorer inhibition on short (≤10 amino acids) compared with long (>20 amino acids) substrates suggested that pyrogallol and luteolin may bind to exosites which are not engaged by the former, although their broad inhibition of MMPs contradicts this hypothesis. It is likely that these molecules, devoid of an obvious ZBG, bind to subsites within the Mp domain of ADAMTS4 and ADAMTS5 rather than *bona fide* exosites.

Luteolin (Figure 16), a flavonoid widely distributed in plants, especially in celery and green pepper, inhibited aggrecanase activity of ADAMTS4 and ADAMTS5, although was only modestly selective over MMPs.^135^ Luteolin effectively inhibited the release of GAGs and ARGS-aggrecan fragments in mouse chondrogenic ATDC5 cells and in murine cartilage explants stimulated with IL-1α/retinoic acid, while MMP aggrecanolytic activity was not affected. Interestingly, this inhibitory effect was partly caused by a transcriptional downregulation of *Adamts4* and *Adamts5* expression, another example of a dual mode of inhibition.

Overall, these findings suggest that the structure of flavonoids should be further modified to improve inhibitory potency and selectivity before these molecules could be tested in clinical studies.

### 3.4 Aptamers

Nucleic acid aptamers, often termed “chemical antibodies”, are short, single-stranded DNA or RNA molecules (20–100 nucleotides in length) that share with antibodies the ability to recognize their targets with exquisite affinity and selectivity.^136^ Complementary base pairing allows the formation of unique 3D folds that can be selected for their ability to bind a specific target through *in vitro* selection methods such as systemic evolution of ligands by exponential enrichment (SELEX). Compared to mAbs, aptamers have theoretically a competitive advantage for therapeutic purposes due to their smaller size (6–30 kDa), lower manufacturing costs, and lower immunogenicity, although they suffer from limited half-life *in vivo* (∼10 min in the absence of specific modifications).^136^ RBM-010 (patents US20210246451A1, WO2019093497) is the first RNA aptamer-based ADAMTS5 inhibitor developed by Ribomic Inc. and is currently in preclinical evaluations.

DNA aptamers are more stable and easier to synthesize compared with RNA aptamers, while RNA aptamers are typically endowed with higher affinity and selectivity.^137^ Yu et al. used SELEX to isolate two DNA aptamers, apt21 and apt25, against ADAMTS5.^138^ Although the two aptamers had affinities in the low nanomolar range (1.54 and 1.79 nM, respectively) they exhibited a poor inhibitory activity in a QF-peptide cleavage assay (52.76 and 61.14 μM, respectively). Inhibition of proteoglycan cleavage was not tested.

Although aptamers have been so far superseded by mAbs in therapeutic applications,^136^ it is likely that more of them will reach the clinic, therefore we expect that R&D investments in aptamer-based aggrecanase inhibitors will grow, albeit at a slow pace.

### 3.5 Peptide-Based Inhibitors

Like protein-based inhibitors, peptide-based inhibitors bind to their targets with an extended surface of interaction, thus generally achieving higher selectivity. However, like small molecules, peptides can be synthesized chemically and are thus cheaper to produce than recombinant proteins. Other advantages include low toxicity and reduced antigenicity.^139^ Therefore, peptide-based inhibitors are potentially endowed with the advantages of the two different classes of molecules. However, due to their small size, peptide-based inhibitors have reduced half-life, an issue that can be addressed in a similar way as TIMPs. So far, few peptide-based aggrecanase inhibitors have been reported, all of them targeting ADAMTS4 (Table 3). Unfortunately, none of them has been tested neither against ADAMTS5, nor against any other metalloproteinase.

**Table 3:** IC_50_ Values (μM) for Inhibition of Aggrecanases by Synthetic Peptides^a^

peptide	parental sequence	ADAMTS4	ADAMTS5	ref
^521^GGWGPWGPWGD^531^	ADAMTS4	17^b^	ND	(140)
^521^GGWGPWGPWGDCSRTCGGG^539^	ADAMTS4	3^b^	ND	(140)
^533^SRTCGGGVQFSSRDCTRPV^551^	ADAMTS4	70^b^	ND	(140)
^555^GGKYCEGRRTRFSCNTEDCP^575^	ADAMTS4	38^b^	ND	(140)
Ac-NEFRQRETYMVF-NH_2_	NA	35^c^	ND	(141)
Ac-DVQEFRGVTAVIR-NH_2_	NA	35^c^	ND	(141)
Ac-DVQ(dE)FRGVTAVIR	NA	10^c^	ND	(141)
KHN(dE)FRQRETYMVFKGK	NA	8^c^	ND	(141)
CASESLC linear	TIMP3	(74)	ND	(142)
CASESLC cyclic	TIMP3	(25)	ND	(142)
CTEASESLAGC linear	TIMP3	(120)	ND	(142)
CTEASESLAGC cyclic	TIMP3	(18)	ND	(142)
CEASESLAGC linear	TIMP3	(34)	ND	(142)
CEASESLAGC cyclic	TIMP3	(3.7)	ND	(142)

a Note that different forms of enzymes were tested in the different studies. *K*_d_ values (in μM) are reported within parentheses and were measured by fluorescence polarization. Abbreviations: ND, not determined; NA, not applicable; Ac, acetyl. Unless indicated differently, all sequences are reported from the N- terminus to the C-terminus.

b Values determined using bovine aggrecan (Glu392-Ala393 cleavage site).

c Values determined using a QF-peptide.

Following the observation that removal of the TS-1 motif greatly reduced the aggrecanase activity of ADAMTS4, Tortorella et al. hypothesized that this domain was involved in aggrecan binding.^140^ They then tested a series of overlapping peptides based on the TS-1 sequence for their ability to inhibit ADAMTS4 aggrecanase activity. These peptides inhibited ADAMTS4 with IC_50_ values in the micromolar range, presumably by competing with ADAMTS4 for binding to aggrecan (Table 3).^140^

Hills et al. reported several peptides inhibiting ADAMTS4 peptidolytic activity with IC_50_ values in the micromolar range (Table 3).^141^ The sequences of these peptides were based on peptide substrates identified by phage display selection of a library of 10^8^ random 13-amino-acid peptides. The amino acid composition of these peptides was equimolar for all 20 amino acids except cysteine.

In an alternative approach, Zhang et al. generated disulfide-bonded cyclic peptides based on the sequence of a short inhibitory loop ^85^EASESLC^91^ (Uniprot ID P35625) of TIMP3.^142^ While the linear peptide bound ADAMTS4 with a weak affinity (74 μM), cyclization improved considerably the affinity by minimizing the entropy penalty of the interaction (Table 3).

Overall, from the few examples reported in the literature it seems that the pharmacological potential of peptide-based inhibitors is far from being unlocked.

### 3.6 Monoclonal Antibodies

mAbs are potent and selective binders of many biologically relevant targets. For this reason, they are well established as therapeutic agents for several diseases including cancer, autoimmune disorders, and infectious diseases (the 100th mAb was approved by the U.S. Food and Drug Administration in 2021).^143^ In 1975, Köhler and Milstein described hybridoma technology, a method to generate mAbs based on the fusion of B-lymphocytes from an immunized animal with immortal myeloma cells.^144^ Soon this method became popular for generation of mAbs for a variety of applications. An alternative way to generate mAbs is phage display, which has superseded hybridoma technology through the creation of large natural and synthetic *in vitro* repertoires of antibody fragments.^145^ Both approaches have been used to generate potent and selective inhibitors of aggrecanases.^146^ The versatility of phage display offers the opportunity to isolate mAbs with desired properties. For example, phage display selections where the active site of ADAMTS5 was blocked with the zinc-chelating inhibitor GM6001 have been used to obtain mAbs targeting ADAMTS5 exosites.^27^ The two most potent inhibitors, 2D3 and 2B9, bound to the Mp/Dis and Sp domains, respectively (Table 4). Competition surface plasmon resonance experiments with TIMP3 and GM6001 confirmed that all these mAbs recognized epitopes outside the active-site cleft. Remarkably, the anti-Sp mAb 2B9 showed inhibitory activity on protein substrates such as aggrecan^27^ and versican^22^ but was unable to inhibit cleavage of a QF-peptide (a clear-cut example of exosite inhibition), while the anti-Mp/Dis mAb 2D3 was able to inhibit efficiently cleavage of both protein and peptide substrates by targeting an epitope in the Dis domain. 2D3 showed potent inhibitory activity of aggrecanase activity in unstimulated human chondrocyte monolayer cultures from healthy donors^27^ and OA cartilage explants.^28^ These mAbs showed exquisite selectivity, with no inhibition observed on ADAMTS4 at concentrations up to 500 nM.^27^ Another anti-ADAMTS5 Sp mAb, CRB0017, developed by Rottapharm using a proprietary selection technology, was effective in delaying cartilage degradation in STR/Ort mice.^147^ Phage display was instead used to isolate an anti-ADAMTS4/ADAMTS5 inhibitory Fab fragment, 237-53, binding to an epitope in the central TS-1 motif of both aggrecanases.^148^ This mAb completely inhibited ADAMTS4 but showed only partial inhibition of ADAMTS5 at a 1:5 enzyme/mAb ratio.

**Table 4:** Properties of Inhibitory mAbs against Aggrecanases^a^

mAb	format	target	epitope	*K*_D_ (nM)	IC_50_^1^ (nM)	ref
7E8.1E3	IgG	ADAMTS4	Mp/Dis	0.25	0.035	(26)
7C7.1H1	IgG	ADAMTS4	CR/Sp	0.29	0.048	(26)
GSK2394000	IgG	ADAMTS5	Mp/Dis	0.21	11	(26)
GSK2394002	IgG	ADAMTS5	Mp/Dis	0.038	0.083	(26)
2B9	scFc-Fv	ADAMTS5	Sp	6.6	90–140	(27)
2D3	scFc-Fv	ADAMTS5	Mp/Dis	3.9	2.5	(27)
					8–90	(28)
1B7	scFc-Fv	ADAMTS5	Mp/Dis	70	NI	(29)
CRB0017	IgG	ADAMTS5	Sp	2.2	NR	(147)
237-53	Fab	ADAMTS4/ADAMTS5	TS-1	12 (ADAMTS4)	80	(148)
				1.5 (ADAMTS5)	NR	
M6495	Bivalent Nb	ADAMTS5	Mp/Dis	0.0037	NR	(150)

a Values determined using aggrecan, Abbreviations: Nb, nanobody, single variable domain derived from heavy-chain-only antibodies of Camelidae; NI, not inhibiting; NR, not reported; scFv-Fc, single-chain variable fragment fused to the immunoglobulin crystallizable fragment.

Several mAbs have been generated by GSK against ADAMTS4 and ADAMTS5. These mAbs showed sub-nanomolar affinity and recognized different domains on their target proteases (Table 4).^26^ Both anti-ADAMTS4 and anti-ADAMTS5 mAbs (670 nM) effectively inhibited the release of aggrecan ARGS-fragments from IL-1β/OSM stimulated human OA cartilage explants, while in the absence of inflammatory stimuli only the anti-ADAMTS5 mAbs were effective. At 10–16 mg/kg, anti-ADAMTS5 mAbs conferred significant protection in the DMM mouse model.^26,31^ Remarkably, intense knee staining was observed 4 days after administration via intraperitoneal injection, thus demonstrating high target engagement.^26^ Prophylactic or therapeutic treatment (10 mg/kg) also protected from mechanical allodynia.^26,31^ These promising results prompted further investigations in a non-human primate model. Administration in cynomolgus monkeys of anti-ADAMTS5 mAb GSK2394002 significantly decreased serum aggrecan ARGS levels. However, sub-endocardial hemorrhage as well a sustained increase in mean arterial pressure and ST segment elevation were observed with doses from 3 to >30 mg/kg, and these side effects were sustained for up to 8 months following a single dose of mAb.^149^ It has been suggested that these cardiovascular effects may be due to inhibition of ADAMTS5 versicanase activity.^149^ Although ADAMTS5 is ∼18-fold more potent than ADAMTS4 as a versicanase *in vitro*,^22^ no mechanistic link between the cardiovascular anomalies elicited by GSK2394002 and ADAMTS5 versicanase activity has been reported so far. However, there are indications that these potentially concerning side effects may be mAb-specific, since another anti-ADAMTS5 mAb, M6495, was safely tolerated in phase I clinical trials.

M6495 is a bivalent nanobody developed by Nordic Bioscience, Merck, and Ablynx, comprising two variable domains sequences derived from llama antibodies separated by a flexible glicyine-serine linker: an N-terminal sequence recognizing ADAMTS5 and a C-terminal sequence binding to human serum albumin to increase its half-life.^150^ M6495 not only inhibited aggrecan degradation in OA synovial membranes, but also decreased Toll-like receptor 2 activation, suggesting a potential application as a painkiller.^151^ Inhibition of ADAMTS5 activity by M6495 decreased the release of a 32-mer aggrecan fragment (generated following independent cleavage by aggrecanases at Glu392-Ala393 and MMPs at N360-F361) which acts as a matrikine by exciting dorsal root ganglion nociceptive neurons in chondrocytes.^152^ Two phase I clinical trials (NCT03583346 and NCT03224702) have been completed for M6495; at least in one of them (NCT03224702), M6495 was safely tolerated at doses up to 300 mg: a single dose of 300 mg resulted in a 45% decrease in circulating ARGS aggrecan levels that was maintained up to 74 days.^153^

## 4 Conclusions and Perspectives

The socioeconomic burden of OA is likely to increase, given the combined trends of aging and rising epidemic of obesity. Despite massive efforts in R&D pipelines, approval of a DMOAD is still far away. More than 20 years after the identification of ADAMTS4 and ADAMTS5 as the aggrecanases involved in cartilage degradation,^17,18^ no molecule able to inhibit their activity has reached the clinic (Table 5).

**Table 5:** Major OA Clinical Trials Investigating Aggrecanase Inhibitors^a^

compound	class	developed by	clinical phase	route of administration	ID	status
**20** (AGG-523)	small molecule/zinc-chelating	Wyeth (now Pfizer)	I (OA)	oral	NCT00427687	completed
			I (knee OA)	oral	NCT00454298	completed
			I (healthy)	oral	NCT00434785	completed
			I (knee OA/healthy)	oral	NCT00380900	completed
			I (healthy)	oral	NCT00369304	completed
**29** (GLPG1972/S201086)	small molecule/zinc-chelating	Galapagos NV	I (healthy)	oral	NCT02612246	completed
			I (OA)	oral	NCT03311009	completed
			II (knee OA)	oral	NCT03595618	completed
			I (healthy)	oral	NCT03143725	completed
			I (healthy)	oral/IV	NCT04136327	completed
			I (healthy)	oral	NCT02851485	completed
			I (healthy)	oral	NCT04137341	completed
CaPPs	sulfated GAGs/exosite	Paradigm Biopharmaceuticals USA (INC)	III (knee OA)	SC	NCT04814719	not yet recruiting
			II/III (knee OA)	SC	NCT04809376	recruiting
M6495	mAb^b^	Nordic Bioscience,	I (knee OA)	SC	NCT03583346	completed
		Merck, and Ablynx	I (healthy)	SC	NCT03224702	completed

a Abbreviations: ID, ClinicalTrials.gov identifier; IV, intravenous; SC, subcutaneous.

b Mechanism of inhibition not reported.

The development of many aggrecanase inhibitors was terminated owing to a lack of efficacy in animal models, which incompletely recapitulate human OA and are therefore poorly predictive of its progression. The majority of these preclinical studies used rodents as model organisms and mimicked mechanical loading/trauma (surgical models such as the DMM model), inflammation (such as the antigen-induced arthritis models) or genetically susceptible joint degeneration (the STR/Ort model).^154^ As discussed in the previous sections, the converse is also true, with several inhibitors showing efficacy in animal models being terminated because of a lack of efficacy in clinical trials. Rodents differ from humans in articular cartilage physiology, weight bearing, gait, and sex-dependent responses to catabolic stimuli and pain.^154^ Developing animal models able to capture more closely the complexity of human OA will help focusing drug development efforts on *bona fide* DMOAD candidates. Another factor that may affect the outcome of clinical trials is the choice of primary end point for the study. Radiographic joint space narrowing (i.e., the decrease in joint space width) is the primary structural end point accepted by the European Medicines Agency and the FDA to prove effectiveness of DMOAD candidates, but it suffers from many limitations, such as the need for long-term follow-up to observe changes in disease progression and its poor applicability to early OA.^155^ To address this, composite end point approaches have been proposed.^156^ However, as noted in a recent FDA draft guidance, “the ability of treatment effects on common measures of structural progression to reliably predict treatment effects on direct measures of how patients function and feel has not been established.”^7^

Finally, the delivery route for the candidate DMOAD should be carefully selected. Intra-articular injections are inconvenient, uncomfortable to the patient and require trained healthcare staff. For these reasons, the ultimate goal for DMOADs has been oral administration. To enhance cartilage penetration, candidate DMOADs can be designed to target chondrocytes or the cartilage ECM. For example, addition of positively charged groups may increase the affinity for negatively charged ECM components such as aggrecan. However, even in the event that these modifications do not severely affect the physicochemical properties of the drug or its ability to effectively engage the target, levels of many proteoglycans such as aggrecan are known to increase in a variety of cardiovascular diseases,^158^ which represent common co-morbidities for OA patients.^159^ Targeting proteoglycans to deliver candidate DMOADs specifically to the cartilage will be a daunting task.

Initially, synthetic chemistry approaches followed in the path marked by MMP inhibitors, a choice that with the hindsight was doomed to failure. These first generation aggrecanase inhibitors predominantly contained the hydroxamate as a ZBG and peptide/peptidomimetic backbone which made them poorly selective. The MSK symptoms exhibited by this class of molecules during previous clinical trials for cancer therapy was a cause of concern.^15^ Because of chronic administration in an older population with multiple co-morbidities, DMOADs must be able to demonstrate utmost safety. Molecules with alternative ZBGs, such as hydantoin GLPG1972/S201086 (**29**),^81^ showed improved selectivity which can be reflected in their safe profile in preclinical and clinical trials (Table 5). Given these first results, further studies involving small-molecule inhibitors bearing this ZBG may be considered as a promising strategy to develop new chemical probes or therapeutic agents.

So far, no zinc-binding inhibitor has received approval from regulatory bodies. There is still the possibility that the presence of ZBG may confer an intrinsic disadvantage to this class of molecules, for example by binding other metzincins. On the other hand, exosite molecules such as sulfated GAGs, flavonoids, and glycoconjugates at the moment do not have the potency required for being tested as feasible DMOADs. It seems obvious that an aggrecanase inhibitor must be endowed with the right combination of selectivity and potency in order to be developed as a DMOAD. Another consideration is that targeting either ADAMTS4 or ADAMTS5 may be preferable to avoid unwanted systemic effects. On this regard, the biological function of ADAMTS5 in tissues other than cartilage still needs to be fully elucidated. The highly selective anti-ADAMTS5 mAb GSK2394002 showed cardiovascular side effects in a non-human primate model^149^ that arrested its progress to the clinic. OA is associated with a slightly increased risk of cardiovascular death compared with non-OA controls,^159,160^ therefore cardiovascular integrity upon DMOAD administration must be preserved. As discussed in section 3.6, significant side effects were not observed for another anti-ADAMTS5 mAb, M6495,^153^ thus further highlighting the extremely complex drug-specific pathways of target engagement. Although classical immunoglobulins remain promising DMOADs, antibody fragments such as nanobodies may have a competitive advantage in terms of target engagement and side effects.

It is worth highlighting that the majority of the anti-aggrecanase mAbs reported so far either block access of substrates to the active-site cleft, for example by “freezing” their target protease in a closed conformation (GSK2394002)^26^ or by binding to exosites in the Dis domain (2D3).^27^ Alternatively, mAbs can block exosites in distal ancillary domains (2B9);^27^ the modality of action of these mAbs resembles those of autoantibodies against ADAMTS13 that mainly target the Sp domain.^161^ Unfortunately, exosite inhibitors have not yet fulfilled their mission. The fact that the anti-Sp mAb 2B9 inhibits not only the aggrecanase, but also the versicanase activity of ADAMTS5^22^ may be a potential red flag for those who hope that these molecules may be able to achieve substrate-specific inhibition, even if such an effect is desirable from a therapeutic point of view.

Another unexplored mechanism by which mAbs can inhibit aggrecanases is by targeting their zymogen activation, as demonstrated with a mAb inhibiting activation of urokinase-type plasminogen activator (uPA).^162^ Exploring alternative approaches is essential not only to enhance the chances of success in our quest for a clinically approved DMOAD, but also to deepen our understanding of aggrecanase biology. Engineering the structure of the endogenous aggrecanase inhibitor TIMP3 has resulted in a number of recombinant variants with increased selectivity and half-life (Table 2). An alternative scaffold for the generation of protein-based inhibitors may be α_2_-macroglobulin (α2M), a 720 kDa homotetrameric plasma inhibitor of a variety of proteases.^163^ α2M is characterized by a unique mechanism of inhibition. Proteolytic cleavage within a bait region 39 residues-long triggers a conformational change resulting in sequestration of the target protein.^163^ Engineering of the bait region generated α2M variants selective for MMP2,^164^ therefore it may be feasible to fine-tune this sequence to target either one or both aggrecanases. Peptide and aptamer inhibitors can also probe unexplored regions in the 3D landscape of ADAMTS4/ADAMTS5 binding sequences, although the reduced number of FDA-approved drugs falling within these categories definitely provides an obstacle for pharmaceutical investments in this field.

Notwithstanding the recent frustrating outcomes of anti-aggrecanase clinical trials, there is room for optimism. Compared to 20 years ago, our knowledge of aggrecanase biology has vastly improved. Not only has the cardiovascular role of these proteases been uncovered^165,166^ but the first exosite sequences have been identified.^22,51^ Further research in the structure and function of aggrecanases will definitely improve our chances to target these elusive proteases for OA therapy.

## Abbreviations Used

ADAM: A Disintegrin and Metalloproteinase
ADAMTS: A Disintegrin and Metalloproteinase with Thrombospondin Motifs
α2M: α_2_-macroglobulin
CaPPS: calcium pentosan polysulfate
CysR: cysteine-rich domain
Dis: dintegrin-like domain
DMM: destabilization of medial meniscus
DMOAD: disease-modifying OA drug
ECG: epicatechin gallate
EGCG: epigallocatechin-3-gallate
ECM: extracellular matrix
GAG: glycosaminoglycan
HTS: high-throughput screening
IL1: interleukin 1
LRP1: low-density lipoprotein receptor-related protein 1
mAb: monoclonal antibody
MMP: matrix metalloproteinase
Mp: metalloproteinase domain
MSK: musculoskeletal
NSAID: non-steroidal anti-inflammatory drug
OSM: oncostatin M
OA: osteoarthritis
PEG: polyethylene glycol
QF: quenched fluorescent
rTIMP3: recombinant TIMP3
SAR: structure–activity relationship
Sp: spacer domain
TIMP: tissue inhibitor of metalloproteinase
TS-1: thrombospondin-type I motif
ZBG: zinc-binding group

## References

[ref1] Hunter D. J.; Bierma-Zeinstra S. Osteoarthritis. Lancet 2019, 393, 1745–1759. 10.1016/S0140-6736(19)30417-9. 31034380

[ref2] Leifer V. P.; Katz J. N.; Losina E. The burden of OA-health services and economics. Osteoarthritis Cartilage 2022, 30, 10–16. 10.1016/j.joca.2021.05.007. 34023527 PMC8605034

[ref3] Tonge D. P.; Pearson M. J.; Jones S. W. The hallmarks of osteoarthritis and the potential to develop personalised disease-modifying pharmacological therapeutics. Osteoarthritis Cartilage 2014, 22, 609–621. 10.1016/j.joca.2014.03.004. 24632293

[ref4] Culliford D. J.; Maskell J.; Kiran A.; Judge A.; Javaid M. K.; Cooper C.; Arden N. K. The lifetime risk of total hip and knee arthroplasty: results from the UK general practice research database. Osteoarthritis Cartilage 2012, 20, 519–524. 10.1016/j.joca.2012.02.636. 22395038

[ref5] Savvidou O.; Milonaki M.; Goumenos S.; Flevas D.; Papagelopoulos P.; Moutsatsou P. Glucocorticoid signaling and osteoarthritis. Mol. Cell. Endocrinol. 2019, 480, 153–166. 10.1016/j.mce.2018.11.001. 30445185

[ref6] Latourte A.; Kloppenburg M.; Richette P. Emerging pharmaceutical therapies for osteoarthritis. Nat. Rev. Rheumatol. 2020, 16, 673–688. 10.1038/s41584-020-00518-6. 33122845

[ref7] U.S. Food and Drug Administration. Osteoarthritis: structural endpoints for the development of drugs, 2018. https://www.fda.gov/regulatory-information/search-fda-guidance-documents/osteoarthritis-structural-endpoints-development-drugs (accessed July 11, 2022).

[ref8] Heinegård D.; Saxne T. The role of the cartilage matrix in osteoarthritis. Nat. Rev. Rheumatol. 2011, 7, 50–56. 10.1038/nrrheum.2010.198. 21119607

[ref9] Cho Y.; Jeong S.; Kim H.; Kang D.; Lee J.; Kang S. B.; Kim J. H. Disease-modifying therapeutic strategies in osteoarthritis: current status and future directions. Exp Mol. Med. 2021, 53, 1689–1696. 10.1038/s12276-021-00710-y. 34848838 PMC8640059

[ref10] Sanchez C.; Bay-Jensen A. C.; Pap T.; Dvir-Ginzberg M.; Quasnichka H.; Barrett-Jolley R.; Mobasheri A.; Henrotin Y. Chondrocyte secretome: a source of novel insights and exploratory biomarkers of osteoarthritis. Osteoarthritis Cartilage 2017, 25, 1199–1209. 10.1016/j.joca.2017.02.797. 28232143

[ref11] Lai W. M.; Hou J. S.; Mow V. C. A triphasic theory for the swelling and deformation behaviors of articular cartilage. J. Biomech Eng. 1991, 113, 245–258. 10.1115/1.2894880. 1921350

[ref12] Zimmerman B. K.; Nims R. J.; Chen A.; Hung C. T.; Ateshian G. A. Direct osmotic pressure measurements in articular cartilage demonstrate nonideal and concentration-dependent phenomena. J. Biomech Eng. 2021, 143, 041007 10.1115/1.4049158. 33210125 PMC7872001

[ref13] Pratta M. A.; Yao W.; Decicco C.; Tortorella M. D.; Liu R. Q.; Copeland R. A.; Magolda R.; Newton R. C.; Trzaskos J. M.; Arner E. C. Aggrecan protects cartilage collagen from proteolytic cleavage. J. Biol. Chem. 2003, 278, 45539–45545. 10.1074/jbc.M303737200. 12890681

[ref14] Karsdal M. A.; Madsen S. H.; Christiansen C.; Henriksen K.; Fosang A. J.; Sondergaard B. C. Cartilage degradation is fully reversible in the presence of aggrecanase but not matrix metalloproteinase activity. Arthritis Res. Ther. 2008, 10, R63 10.1186/ar2434. 18513402 PMC2483454

[ref15] Coussens L. M.; Fingleton B.; Matrisian L. M. Matrix metalloproteinase inhibitors and cancer: trials and tribulations. Science 2002, 295, 2387–2392. 10.1126/science.1067100. 11923519

[ref16] Krzeski P.; Buckland-Wright C.; Bálint G.; Cline G. A.; Stoner K.; Lyon R.; Beary J.; Aronstein W. S.; Spector T. D. Development of musculoskeletal toxicity without clear benefit after administration of PG-116800, a matrix metalloproteinase inhibitor, to patients with knee osteoarthritis: a randomized, 12-month, double-blind, placebo-controlled study. Arthritis Res. Ther. 2007, 9, R109 10.1186/ar2315. 17958901 PMC2212568

[ref17] Tortorella M. D.; Burn T. C.; Pratta M. A.; Abbaszade I.; Hollis J. M.; Liu R.; Rosenfeld S. A.; Copeland R. A.; Decicco C. P.; Wynn R.; Rockwell A.; Yang F.; Duke J. L.; Solomon K.; George H.; Bruckner R.; Nagase H.; Itoh Y.; Ellis D. M.; Ross H.; Wiswall B. H.; Murphy K.; Hillman M. C. Jr; Hollis G. F.; Newton R. C.; Magolda R. L.; Trzaskos J. M.; Arner E. C. Purification and cloning of aggrecanase-1: a member of the ADAMTS family of proteins. Science 1999, 284, 1664–1666. 10.1126/science.284.5420.1664. 10356395

[ref18] Abbaszade I.; Liu R. Q.; Yang F.; Rosenfeld S. A.; Ross O. H.; Link J. R.; Ellis D. M.; Tortorella M. D.; Pratta M. A.; Hollis J. M.; Wynn R.; Duke J. L.; George H. J.; Hillman M. C. Jr; Murphy K.; Wiswall B. H.; Copeland R. A.; Decicco C. P.; Bruckner R.; Nagase H.; Itoh H.; Newton R. C.; Magolda R. L.; Trzaskos J. M.; Hollis G. F.; Arner E. C.; Burn T. C. Cloning and characterization of ADAMTS11, an aggrecanase from the ADAMTS family. J. Biol. Chem. 1999, 274, 23443–23450. 10.1074/jbc.274.33.23443. 10438522

[ref19] Santamaria S. ADAMTS-5: A difficult teenager turning 20. Int. J. Exp. Pathol. 2020, 101, 4–20. 10.1111/iep.12344. 32219922 PMC7306899

[ref20] Gendron C.; Kashiwagi M.; Lim N. H.; Enghild J. J.; Thøgersen I. B.; Hughes C.; Caterson B.; Nagase H. Proteolytic activities of human ADAMTS-5: comparative studies with ADAMTS-4. J. Biol. Chem. 2007, 282, 18294–18306. 10.1074/jbc.M701523200. 17430884

[ref21] Fushimi K.; Troeberg L.; Nakamura H.; Lim N. H.; Nagase H. Functional differences of the catalytic and non-catalytic domains in human ADAMTS-4 and ADAMTS-5 in aggrecanolytic activity. J. Biol. Chem. 2008, 283, 6706–6716. 10.1074/jbc.M708647200. 18156631

[ref22] Santamaria S.; Yamamoto K.; Teraz-Orosz A.; Koch C.; Apte S. S.; de Groot R.; Lane D. A.; Ahnström J. Exosites in hypervariable loops of ADAMTS spacer domains control substrate recognition and proteolysis. Sci. Rep. 2019, 9, 10914 10.1038/s41598-019-47494-w. 31358852 PMC6662762

[ref23] Glasson S. S.; Askew R.; Sheppard B.; Carito B. A.; Blanchet T.; Ma H. L.; Flannery C. R.; Kanki K.; Wang E.; Peluso D.; Yang Z.; Majumdar M. K.; Morris E. A. Characterization of and osteoarthritis susceptibility in ADAMTS-4-knockout mice. Arthritis Rheum. 2004, 50, 2547–2558. 10.1002/art.20558. 15334469

[ref24] Glasson S. S.; Askew R.; Sheppard B.; Carito B.; Blanchet T.; Ma H. L.; Flannery C. R.; Peluso D.; Kanki K.; Yang Z.; Majumdar M. K.; Morris E. A. Deletion of active ADAMTS5 prevents cartilage degradation in a murine model of osteoarthritis. Nature 2005, 434, 644–648. 10.1038/nature03369. 15800624

[ref25] Stanton H.; Rogerson F. M.; East C. J.; Golub S. B.; Lawlor K. E.; Meeker C. T.; Little C. B.; Last K.; Farmer P. J.; Campbell I. K.; Fourie A. M.; Fosang A. J. ADAMTS5 is the major aggrecanase in mouse cartilage in vivo and in vitro. Nature 2005, 434, 648–652. 10.1038/nature03417. 15800625

[ref26] Larkin J.; Lohr T. A.; Elefante L.; Shearin J.; Matico R.; Su J. L.; Xue Y.; Liu F.; Genell C.; Miller R. E.; Tran P. B.; Malfait A. M.; Maier C. C.; Matheny C. J. Translational development of an ADAMTS-5 antibody for osteoarthritis disease modification. Osteoarthritis Cartilage 2015, 23, 1254–1266. 10.1016/j.joca.2015.02.778. 25800415 PMC4516626

[ref27] Santamaria S.; Yamamoto K.; Botkjaer K.; Tape C.; Dyson M. R.; McCafferty J.; Murphy G.; Nagase H. Antibody-based exosite inhibitors of ADAMTS-5 (aggrecanase-2). Biochem. J. 2015, 471, 391–401. 10.1042/BJ20150758. 26303525 PMC4613496

[ref28] Yamamoto K.; Santamaria S.; Botkjaer K. A.; Dudhia J.; Troeberg L.; Itoh Y.; Murphy G.; Nagase H. Inhibition of shedding of low-density lipoprotein receptor-related protein 1 reverses cartilage matrix degradation in osteoarthritis. Arthritis Rheumatol. 2017, 69, 1246–1256. 10.1002/art.40080. 28235248 PMC5449214

[ref29] Santamaria S.; Fedorov O.; McCafferty J.; Murphy G.; Dudhia J.; Nagase H.; Yamamoto K. Development of a monoclonal anti-ADAMTS-5 antibody that specifically blocks the interaction with LRP1. MAbs 2017, 9, 595–602. 10.1080/19420862.2017.1304341. 28306378 PMC5419085

[ref30] Malfait A. M.; Ritchie J.; Gil A. S.; Austin J. S.; Hartke J.; Qin W.; Tortorella M. D.; Mogil J. S. ADAMTS-5 deficient mice do not develop mechanical allodynia associated with osteoarthritis following medial meniscal destabilization. Osteoarthritis Cartilage 2010, 18, 572–580. 10.1016/j.joca.2009.11.013. 20036347

[ref31] Miller R. E.; Tran P. B.; Ishihara S.; Larkin J.; Malfait A. M. Therapeutic effects of an anti-ADAMTS-5 antibody on joint damage and mechanical allodynia in a murine model of osteoarthritis. Osteoarthritis Cartilage 2016, 24, 299–306. 10.1016/j.joca.2015.09.005. 26410555 PMC4743933

[ref32] Bondeson J.; Wainwright S.; Hughes C.; Caterson B. The regulation of the ADAMTS4 and ADAMTS5 aggrecanases in osteoarthritis: a review. Clin. Exp. Rheumatol. 2008, 26, 139–145. 18328163

[ref33] Inagaki J.; Nakano A.; Hatipoglu O. F.; Ooka Y.; Tani Y.; Miki A.; Ikemura K.; Opoku G.; Ando R.; Kodama S.; Ohtsuki T.; Yamaji H.; Yamamoto S.; Katsuyama E.; Watanabe S.; Hirohata S. Potential of a novel chemical compound targeting matrix metalloprotease-13 for early osteoarthritis: An In Vitro Study. Int. J. Mol. Sci. 2022, 23, 2681 10.3390/ijms23052681. 35269821 PMC8910651

[ref34] Chu X.; You H.; Yuan X.; Zhao W.; Li W.; Guo X. Protective effect of lentivirus-mediated siRNA targeting ADAMTS-5 on cartilage degradation in a rat model of osteoarthritis. Int. J. Mol. Med. 2013, 31, 1222–1228. 10.3892/ijmm.2013.1318. 23546441

[ref35] Hoshi H.; Akagi R.; Yamaguchi S.; Muramatsu Y.; Akatsu Y.; Yamamoto Y.; Sasaki T.; Takahashi K.; Sasho T. Effect of inhibiting MMP13 and ADAMTS5 by intra-articular injection of small interfering RNA in a surgically induced osteoarthritis model of mice. Cell Tissue Res. 2017, 368, 379–387. 10.1007/s00441-016-2563-y. 28120109

[ref36] Bode W.; Gomis-Rüth F. X.; Stöckler W. Astacins, serralysins, snake venom and matrix metalloproteinases exhibit identical zinc-binding environments (HEXXHXXGXXH and Met-turn) and topologies and should be grouped into a common family, the ’metzincins’. FEBS Lett. 1993, 331, 134–140. 10.1016/0014-5793(93)80312-I. 8405391

[ref37] Van Wart H. E.; Birkedal-Hansen H. The cysteine switch: a principle of regulation of metalloproteinase activity with potential applicability to the entire matrix metalloproteinase gene family. Proc. Natl. Acad. Sci. U.S.A. 1990, 87, 5578–5582. 10.1073/pnas.87.14.5578. 2164689 PMC54368

[ref38] Gao G.; Westling J.; Thompson V. P.; Howell T. D.; Gottschall P. E.; Sandy J. D. Activation of the proteolytic activity of ADAMTS4 (aggrecanase-1) by C-terminal truncation. J. Biol. Chem. 2002, 277, 11034–11041. 10.1074/jbc.M107443200. 11796708

[ref39] Malfait A. M.; Arner E. C.; Song R. H.; Alston J. T.; Markosyan S.; Staten N.; Yang Z.; Griggs D. W.; Tortorella M. D. Proprotein convertase activation of aggrecanases in cartilage in situ. Arch. Biochem. Biophys. 2008, 478, 43–51. 10.1016/j.abb.2008.07.012. 18671934

[ref40] Longpré J. M.; McCulloch D. R.; Koo B. H.; Alexander J. P.; Apte S. S.; Leduc R. Characterization of proADAMTS5 processing by proprotein convertases. Int. J. Biochem. Cell Biol. 2009, 41, 1116–1126. 10.1016/j.biocel.2008.10.008. 18992360

[ref41] Mosyak L.; Georgiadis K.; Shane T.; Svenson K.; Hebert T.; McDonagh T.; Mackie S.; Olland S.; Lin L.; Zhong X.; Kriz R.; Reifenberg E. L.; Collins-Racie L. A.; Corcoran C.; Freeman B.; Zollner R.; Marvell T.; Vera M.; Sum P.-E.; Lavallie E. R.; Stahl M.; Somers W. Crystal structures of the two major aggrecan degrading enzymes, ADAMTS4 and ADAMTS5. Protein Sci. 2008, 17, 16–21. 10.1110/ps.073287008. 18042673 PMC2144589

[ref42] Shieh H. S.; Mathis K. J.; Williams J. M.; Hills R. L.; Wiese J. F.; Benson T. E.; Kiefer J. R.; Marino M. H.; Carroll J. N.; Leone J. W.; Malfait A. M.; Arner E. C.; Tortorella M. D.; Tomasselli A. High resolution crystal structure of the catalytic domain of ADAMTS-5 (aggrecanase-2). J. Biol. Chem. 2008, 283, 1501–1507. 10.1074/jbc.M705879200. 17991750

[ref43] Lill M. A.; Danielson M. L. Computer-aided drug design platform using PyMOL. J. Comput. Aided Mol. Des. 2011, 25, 13–19. 10.1007/s10822-010-9395-8. 21053052

[ref44] Ciccone L.; Policar C.; Stura E. A.; Shepard W. Human TTR conformation altered by rhenium tris-carbonyl derivatives. J. Struct. Biol. 2016, 195, 353–364. 10.1016/j.jsb.2016.07.002. 27402536

[ref45] Polsinelli I.; Nencetti S.; Shepard W.; Ciccone L.; Orlandini E.; Stura E. A. A new crystal form of human transthyretin obtained with a curcumin derived ligand. J. Struct Biol. 2016, 194, 8–17. 10.1016/j.jsb.2016.01.007. 26796656

[ref46] Solomon R. W. Free and open source software for the manipulation of digital images. AJR Am. J. Roentgenol. 2009, 192, W330–W334. 10.2214/AJR.08.2190. 19457798

[ref47] Schechter I.; Berger A. On the size of the active site in proteases. I. Papain. Biochem. Biophys. Res. Commun. 1967, 27, 157–162. 10.1016/S0006-291X(67)80055-X. 6035483

[ref48] Petri A.; Kim H. J.; Xu Y.; de Groot R.; Li C.; Vandenbulcke A.; Vanhoorelbeke K.; Emsley J.; Crawley J. T. B. Crystal structure and substrate-induced activation of ADAMTS13. Nat. Commun. 2019, 10, 3781 10.1038/s41467-019-11474-5. 31439947 PMC6706451

[ref49] Tallant C.; García-Castellanos R.; Baumann U.; Gomis-Rüth F. X. On the relevance of the Met-turn methionine in metzincins. J. Biol. Chem. 2010, 285, 13951–13957. 10.1074/jbc.M109.083378. 20202937 PMC2859557

[ref50] Takeda S. ADAM and ADAMTS family proteins and snake venom metalloproteinases: a structural overview. Toxins (Basel) 2016, 8, 155 10.3390/toxins8050155. PMC4885070 27196928

[ref51] Santamaria S.; Cuffaro D.; Nuti E.; Ciccone L.; Tuccinardi T.; Liva F.; D’Andrea F.; de Groot R.; Rossello A.; Ahnström J. Exosite inhibition of ADAMTS-5 by a glycoconjugated arylsulfonamide. Sci. Rep. 2021, 11, 949 10.1038/s41598-020-80294-1. 33441904 PMC7806935

[ref52] de Groot R.; Lane D. A.; Crawley J. T. The role of the ADAMTS13 cysteine-rich domain in VWF binding and proteolysis. Blood 2015, 125, 1968–1975. 10.1182/blood-2014-08-594556. 25564400 PMC4366626

[ref53] Akiyama M.; Takeda S.; Kokame K.; Takagi J.; Miyata T. Crystal structures of the noncatalytic domains of ADAMTS13 reveal multiple discontinuous exosites for von Willebrand factor. Proc. Natl. Acad. Sci. U.S.A. 2009, 106, 19274–19279. 10.1073/pnas.0909755106. 19880749 PMC2780749

[ref54] Jumper J.; Evans R.; Pritzel A.; Green T.; Figurnov M.; Ronneberger O.; Tunyasuvunakool K.; Bates R.; Žídek A.; Potapenko A.; Bridgland A.; Meyer C.; Kohl S.; Ballard A. J.; Cowie A.; Romera-Paredes B.; Nikolov S.; Jain R.; Adler J.; Back T.; Hassabis D.; et al. Highly accurate protein structure prediction with AlphaFold. Nature 2021, 596, 583–589. 10.1038/s41586-021-03819-2. 34265844 PMC8371605

[ref55] Cheng A. C.; Coleman R. G.; Smyth K. T.; Cao Q.; Soulard P.; Caffrey D. R.; Salzberg A. C.; Huang E. S. Structure-based maximal affinity model predicts small-molecule druggability. Nat. Biotechnol. 2007, 25, 71–75. 10.1038/nbt1273. 17211405

[ref56] Troisi R.; Balasco N.; Autiero I.; Vitagliano L.; Sica F. Exosite binding in thrombin: a global structural/dynamic overview of complexes with aptamers and other ligands. Int. J. Mol. Sci. 2021, 22, 10803 10.3390/ijms221910803. 34639143 PMC8509272

[ref57] Saghatelian A.; Jessani N.; Joseph A.; Humphrey M.; Cravatt B. F. Activity-based probes for the proteomic profiling of metalloproteases. Proc. Natl. Acad. Sci. U.S.A. 2004, 101, 10000–10005. 10.1073/pnas.0402784101. 15220480 PMC454150

[ref58] Yao W.; Wasserman Z. R.; Chao M.; Reddy G.; Shi E.; Liu R. Q.; Covington M. B.; Arner E. C.; Pratta M. A.; Tortorella M.; Magolda R. L.; Newton R.; Qian M.; Ribadeneira M. D.; Christ D.; Wexler R. R.; Decicco C. P. Design and synthesis of a series of (2R)-N(4)-hydroxy-2-(3-hydroxybenzyl)-N(1)-[(1S,2R)-2-hydroxy-2,3-dihydro-1H-inden-1yl] butanediamide derivatives as potent, selective, and orally bioavailable aggrecanase inhibitors. J. Med. Chem. 2001, 44, 3347–3350. 10.1021/jm015533c. 11585439

[ref59] Arner E. C.; Decicco C. P.; Cherney R.; Tortorella M. D. Cleavage of native cartilage aggrecan by neutrophil collagenase (MMP-8) is distinct from endogenous cleavage by aggrecanase. J. Biol. Chem. 1997, 272, 9294–9299. 10.1074/jbc.272.14.9294. 9083065

[ref60] Tortorella M. D.; Tomasselli A. G.; Mathis K. J.; Schnute M. E.; Woodard S. S.; Munie G.; Williams J. M.; Caspers N.; Wittwer A. J.; Malfait A. M.; Shieh H. S. Structural and inhibition analysis reveals the mechanism of selectivity of a series of aggrecanase inhibitors. J. Biol. Chem. 2009, 284, 24185–24191. 10.1074/jbc.M109.029116. 19586907 PMC2782012

[ref61] Yao W.; Chao M.; Wasserman Z. R.; Liu R. Q.; Covington M. B.; Newton R.; Christ D.; Wexler R. R.; Decicco C. P. Potent P1′ biphenylmethyl substituted aggrecanase inhibitors. Bioorg. Med. Chem. Lett. 2002, 12, 101–104. 10.1016/S0960-894X(01)00704-1. 11738583

[ref62] Cherney R. J.; Mo R.; Meyer D. T.; Wang L.; Yao W.; Wasserman Z. R.; Liu R. Q.; Covington M. B.; Tortorella M. D.; Arner E. C.; Qian M.; Christ D. D.; Trzaskos J. M.; Newton R. C.; Magolda R. L.; Decicco C. P. Potent and selective aggrecanase inhibitors containing cyclic P1 substituents. Bioorg. Med. Chem. Lett. 2003, 13, 1297–1300. 10.1016/S0960-894X(03)00124-0. 12657268

[ref63] Noe M. C.; Natarajan V.; Snow S. L.; Mitchell P. G.; Lopresti-Morrow L.; Reeves L. M.; Yocum S. A.; Carty T. J.; Barberia J. A.; Sweeney F. J.; Liras J. L.; Vaughn M.; Hardink J. R.; Hawkins J. M.; Tokar C. Discovery of 3,3-dimethyl-5-hydroxypipecolic hydroxamate-based inhibitors of aggrecanase and MMP-13. Bioorg. Med. Chem. Lett. 2005, 15, 2808–2811. 10.1016/j.bmcl.2005.03.105. 15911259

[ref64] Noe M. C.; Natarajan V.; Snow S. L.; Wolf-Gouveia L. A.; Mitchell P. G.; Lopresti-Morrow L.; Reeves L. M.; Yocum S. A.; Otterness I.; Bliven M. A.; Carty T. J.; Barberia J. T.; Sweeney F. J.; Liras J. L.; Vaughn M. Discovery of 3-OH-3-methylpipecolic hydroxamates: potent orally active inhibitors of aggrecanase and MMP-13. Bioorg. Med. Chem. Lett. 2005, 15, 3385–3388. 10.1016/j.bmcl.2005.05.037. 15953722

[ref65] Cappelli A.; Nannicini C.; Valenti S.; Giuliani G.; Anzini M.; Mennuni L.; Giordani A.; Caselli G.; Stasi L. P.; Makovec F.; Giorgi G.; Vomero S. Design, synthesis, and preliminary biological evaluation of pyrrolo[3,4-c]quinolin-1-one and oxoisoindoline derivatives as aggrecanase inhibitors. ChemMedChem 2010, 5, 739–748. 10.1002/cmdc.200900523. 20379990

[ref66] De Savi C.; Pape A.; Cumming J. G.; Ting A.; Smith P. D.; Burrows J. N.; Mills M.; Davies C.; Lamont S.; Milne D.; Cook C.; Moore P.; Sawyer Y.; Gerhardt S. The design and synthesis of novel N-hydroxyformamide inhibitors of ADAM-TS4 for the treatment of osteoarthritis. Bioorg. Med. Chem. Lett. 2011, 21, 1376–1381. 10.1016/j.bmcl.2011.01.036. 21300546

[ref67] De Savi C.; Pape A.; Sawyer Y.; Milne D.; Davies C.; Cumming J. G.; Ting A.; Lamont S.; Smith P. D.; Tart J.; Page K.; Moore P. Orally active achiral N-hydroxyformamide inhibitors of ADAM-TS4 (aggrecanase-1) and ADAM-TS5 (aggrecanase-2) for the treatment of osteoarthritis. Bioorg. Med. Chem. Lett. 2011, 21, 3301–3306. 10.1016/j.bmcl.2011.04.028. 21536437

[ref68] Nuti E.; Santamaria S.; Casalini F.; Yamamoto K.; Marinelli L.; La Pietra V.; Novellino E.; Orlandini E.; Nencetti S.; Marini A. M.; Salerno S.; Taliani S.; Da Settimo F.; Nagase H.; Rossello A. Arylsulfonamide inhibitors of aggrecanases as potential therapeutic agents for osteoarthritis: synthesis and biological evaluation. Eur. J. Med. Chem. 2013, 62, 379–394. 10.1016/j.ejmech.2012.12.058. 23376997

[ref69] Monovich L. G.; Tommasi R. A.; Fujimoto R. A.; Blancuzzi V.; Clark K.; Cornell W. D.; Doti R.; Doughty J.; Fang J.; Farley D.; Fitt J.; Ganu V.; Goldberg R.; Goldstein R.; Lavoie S.; Kulathila R.; Macchia W.; Parker D. T.; Melton R.; O’Byrne E.; Pastor G.; Pellas T.; Quadros E.; Reel N.; Roland D. M.; Sakane Y.; Singh H.; Skiles J.; Somers J.; Toscano K.; Wigg A.; Zhou S.; Zhu L.; Shieh W. C.; Xue S.; McQuire L. W. Discovery of potent, selective, and orally active carboxylic acid based inhibitors of matrix metalloproteinase-13. J. Med. Chem. 2009, 52, 3523–3538. 10.1021/jm801394m. 19422229

[ref70] Xiang J. S.; Hu Y.; Rush T. S.; Thomason J. R.; Ipek M.; Sum P. E.; Abrous L.; Sabatini J. J.; Georgiadis K.; Reifenberg E.; Majumdar M.; Morris E. A.; Tam S. Synthesis and biological evaluation of biphenylsulfonamide carboxylate aggrecanase-1 inhibitors. Bioorg. Med. Chem. Lett. 2006, 16, 311–316. 10.1016/j.bmcl.2005.10.001. 16275085

[ref71] Hopper D. W.; Vera M. D.; How D.; Sabatini J.; Xiang J. S.; Ipek M.; Thomason J.; Hu Y.; Feyfant E.; Wang Q.; Georgiadis K. E.; Reifenberg E.; Sheldon R. T.; Keohan C. C.; Majumdar M. K.; Morris E. A.; Skotnicki J.; Sum P. E. Synthesis and biological evaluation of ((4-keto)-phenoxy)methyl biphenyl-4-sulfonamides: a class of potent aggrecanase-1 inhibitors. Bioorg. Med. Chem. Lett. 2009, 19, 2487–2491. 10.1016/j.bmcl.2009.03.056. 19329309

[ref72] Hu Y.; Xing L.; Thomason J. R.; Xiang J.; Ipek M.; Guler S.; Li H.; Sabatini J.; Chockalingam P.; Reifenberg E.; Sheldon R.; Morris E. A.; Georgiadis K. E.; Tam S. Continued exploration of biphenylsulfonamide scaffold as a platform for aggrecanase-1 inhibition. Bioorg. Med. Chem. Lett. 2011, 21, 6800–6803. 10.1016/j.bmcl.2011.09.036. 21982494

[ref73] Shiozaki M.; Maeda K.; Miura T.; Ogoshi Y.; Haas J.; Fryer A. M.; Laird E. R.; Littmann N. M.; Andrews S. W.; Josey J. A.; Mimura T.; Shinozaki Y.; Yoshiuchi H.; Inaba T. Novel N-substituted 2-phenyl-1-sulfonylamino-cyclopropane carboxylates as selective ADAMTS-5 (Aggrecanase-2) inhibitors. Bioorg. Med. Chem. Lett. 2009, 19, 1575–1580. 10.1016/j.bmcl.2009.02.024. 19243944

[ref74] Shiozaki M.; Maeda K.; Miura T.; Kotoku M.; Yamasaki T.; Matsuda I.; Aoki K.; Yasue K.; Imai H.; Ubukata M.; Suma A.; Yokota M.; Hotta T.; Tanaka M.; Hase Y.; Haas J.; Fryer A. M.; Laird E. R.; Littmann N. M.; Andrews S. W.; Josey J. A.; Mimura T.; Shinozaki Y.; Yoshiuchi H.; Inaba T. Discovery of (1S,2R,3R)-2,3-dimethyl-2-phenyl-1-sulfamidocyclopropanecarboxylates: novel and highly selective aggrecanase inhibitors. J. Med. Chem. 2011, 54, 2839–2863. 10.1021/jm101609j. 21417219

[ref75] Peng L.; Duan L.; Liu X.; Shen M.; Li Y.; Yan J.; Li H.; Ding K. Structure-activity study on a series of α-glutamic acid scaffold based compounds as new ADAMTS inhibitors. Bioorg. Med. Chem. Lett. 2011, 21, 4457–4461. 10.1016/j.bmcl.2011.06.009. 21733683

[ref76] Chockalingam P. S.; Sun W.; Rivera-Bermudez M. A.; Zeng W.; Dufield D. R.; Larsson S.; Lohmander L. S.; Flannery C. R.; Glasson S. S.; Georgiadis K. E.; Morris E. A. Elevated aggrecanase activity in a rat model of joint injury is attenuated by an aggrecanase specific inhibitor. Osteoarthritis Cartilage 2011, 19, 315–323. 10.1016/j.joca.2010.12.004. 21163358

[ref77] Atobe M.; Maekawara N.; Kawanishi M.; Suzuki H.; Tanaka E.; Miyoshi S. Design, synthesis and SAR investigation of thienosultam derivatives as ADAMTS-5 (aggrecanase-2) inhibitors. Bioorg. Med. Chem. Lett. 2013, 23, 2111–2116. 10.1016/j.bmcl.2013.01.120. 23453072

[ref78] Durham T. B.; Klimkowski V. J.; Rito C. J.; Marimuthu J.; Toth J. L.; Liu C.; Durbin J. D.; Stout S. L.; Adams L.; Swearingen C.; Lin C.; Chambers M. G.; Thirunavukkarasu K.; Wiley M. R. Identification of potent and selective hydantoin inhibitors of aggrecanase-1 and aggrecanase-2 that are efficacious in both chemical and surgical models of osteoarthritis. J. Med. Chem. 2014, 57, 10476–10485. 10.1021/jm501522n. 25415648

[ref79] Wiley M. R.; Durham T. B.; Adams L. A.; Chambers M. G.; Lin C.; Liu C.; Marimuthu J.; Mitchell P. G.; Mudra D. R.; Swearingen C. A.; Toth J. L.; Weller J. M.; Thirunavukkarasu K. Use of osmotic pumps to establish the pharmacokinetic-pharmacodynamic relationship and define desirable human performance characteristics for aggrecanase inhibitors. J. Med. Chem. 2016, 59, 5810–5822. 10.1021/acs.jmedchem.6b00398. 27194201

[ref80] Durham T. B.; Marimuthu J.; Toth J. L.; Liu C.; Adams L.; Mudra D. R.; Swearingen C.; Lin C.; Chambers M. G.; Thirunavukkarasu K.; Wiley M. R. A Highly selective hydantoin inhibitor of aggrecanase-1 and aggrecanase-2 with a low projected human dose. J. Med. Chem. 2017, 60, 5933–5939. 10.1021/acs.jmedchem.7b00650. 28613895

[ref81] Brebion F.; Gosmini R.; Deprez P.; Varin M.; Peixoto C.; Alvey L.; Jary H.; Bienvenu N.; Triballeau N.; Blanque R.; Cottereaux C.; Christophe T.; Vandervoort N.; Mollat P.; Touitou R.; Leonard P.; De Ceuninck F.; Botez I.; Monjardet A.; van der Aar E.; Amantini D. Discovery of GLPG1972/S201086, a potent, selective, and orally bioavailable ADAMTS-5 inhibitor for the treatment of osteoarthritis. J. Med. Chem. 2021, 64, 2937–2952. 10.1021/acs.jmedchem.0c02008. 33719441

[ref82] Wittwer A. J.; Hills R. L.; Keith R. H.; Munie G. E.; Arner E. C.; Anglin C. P.; Malfait A. M.; Tortorella M. D. Substrate-dependent inhibition kinetics of an active site-directed inhibitor of ADAMTS-4 (Aggrecanase 1). Biochemistry 2007, 46, 6393–6401. 10.1021/bi7000642. 17487981

[ref83] Clement-Lacroix P.; Little C. B.; Smith M. M.; Cottereaux C.; Merciris D.; Meurisse S.; Mollat P.; Touitou R.; Brebion F.; Gosmini R.; De Ceuninck F.; Botez I.; Lepescheux L.; van der Aar E.; Christophe T.; Vandervoort N.; Blanqué R.; Comas D.; Deprez P.; Amantini D. Pharmacological characterization of GLPG1972/S201086, a potent and selective small-molecule inhibitor of ADAMTS5. Osteoarthritis Cartilage 2022, 30, 291–301. 10.1016/j.joca.2021.08.012. 34626798

[ref84] van der Aar E.; Deckx H.; Dupont S.; Fieuw A.; Delage S.; Larsson S.; Struglics A.; Lohmander L. S.; Lalande A.; Leroux E.; Amantini D.; Passier P. Safety, pharmacokinetics, and pharmacodynamics of the ADAMTS-5 inhibitor GLPG1972/S201086 in healthy volunteers and participants with osteoarthritis of the knee or hip. Clin Pharmacol. Drug Dev. 2022, 11, 112–122. 10.1002/cpdd.1042. 34859612 PMC9299907

[ref85] Bursavich M. G.; Gilbert A. M.; Lombardi S.; Georgiadis K. E.; Reifenberg E.; Flannery C. R.; Morris E. A. Synthesis and evaluation of aryl thioxothiazolidinone inhibitors of ADAMTS-5 (Aggrecanase-2). Bioorg. Med. Chem. Lett. 2007, 17, 1185–1188. 10.1016/j.bmcl.2006.12.027. 17210251

[ref86] Gilbert A. M.; Bursavich M. G.; Lombardi S.; Georgiadis K. E.; Reifenberg E.; Flannery C. R.; Morris E. A. 5-((1H-pyrazol-4-yl)methylene)-2-thioxothiazolidin-4-one inhibitors of ADAMTS-5. Bioorg. Med. Chem. Lett. 2007, 17, 1189–1192. 10.1016/j.bmcl.2006.12.020. 17210252

[ref87] Atobe M.; Maekawara N.; Ishiguro N.; Sogame S.; Suenaga Y.; Kawanishi M.; Suzuki H.; Jinno N.; Tanaka E.; Miyoshi S. A series of thiazole derivatives bearing thiazolidin-4-one as non-competitive ADAMTS-5 (aggrecanase-2) inhibitors. Bioorg. Med. Chem. Lett. 2013, 23, 2106–2110. 10.1016/j.bmcl.2013.01.121. 23453070

[ref88] Copeland R. A. Enzymes: A practical introduction to structure, mechanism, and data Analysis, 2nd ed.; Wiley-VCH: New York, 2000.

[ref89] Sogame S.; Suenaga Y.; Atobe M.; Kawanishi M.; Tanaka E.; Miyoshi S. Discovery of a benzimidazole series of ADAMTS-5 (aggrecanase-2) inhibitors by scaffold hopping. Eur. J. Med. Chem. 2014, 71, 250–258. 10.1016/j.ejmech.2013.10.075. 24316668

[ref90] Maingot L.; Leroux F.; Landry V.; Dumont J.; Nagase H.; Villoutreix B.; Sperandio O.; Deprez-Poulain R.; Deprez B. New non-hydroxamic ADAMTS-5 inhibitors based on the 1,2,4-triazole-3-thiol scaffold. Bioorg. Med. Chem. Lett. 2010, 20, 6213–6216. 10.1016/j.bmcl.2010.08.108. 20846863

[ref91] Maingot L.; Elbakali J.; Dumont J.; Bosc D.; Cousaert N.; Urban A.; Deglane G.; Villoutreix B.; Nagase H.; Sperandio O.; Leroux F.; Deprez B.; Deprez-Poulain R. Aggrecanase-2 inhibitors based on the acylthiosemicarbazide zinc-binding group. Eur. J. Med. Chem. 2013, 69, 244–261. 10.1016/j.ejmech.2013.08.027. 24044937

[ref92] Bursavich M. G.; Gilbert A. M.; Lombardi S.; Georgiadis K. E.; Reifenberg E.; Flannery C. R.; Morris E. A. 5′-Phenyl-3′H-spiro[indoline-3,2′-[1,3,4]thiadiazol]-2-one inhibitors of ADAMTS-5 (aggrecanase-2). Bioorg. Med. Chem. Lett. 2007, 17, 5630–5633. 10.1016/j.bmcl.2007.07.048. 17804234

[ref93] Gilbert A. M.; Bursavich M. G.; Lombardi S.; Georgiadis K. E.; Reifenberg E.; Flannery C. R.; Morris E. A. N-((8-hydroxy-5-substituted-quinolin-7-yl)(phenyl)methyl)-2-phenyloxy/amino-acetamide inhibitors of ADAMTS-5 (Aggrecanase-2). Bioorg. Med. Chem. Lett. 2008, 18, 6454–6457. 10.1016/j.bmcl.2008.10.065. 18974001

[ref94] Deng H.; O’Keefe H.; Davie C. P.; Lind K. E.; Acharya R. A.; Franklin G. J.; Larkin J.; Matico R.; Neeb M.; Thompson M. M.; Lohr T.; Gross J. W.; Centrella P. A.; O’Donovan G. K.; Bedard K. L.; van Vloten K.; Mataruse S.; Skinner S. R.; Belyanskaya S. L.; Carpenter T. Y.; Shearer T. W.; Clark M. A.; Cuozzo J. W.; Arico-Muendel C. C.; Morgan B. A. Discovery of highly potent and selective small molecule ADAMTS-5 inhibitors that inhibit human cartilage degradation via encoded library technology (ELT). J. Med. Chem. 2012, 55, 7061–7079. 10.1021/jm300449x. 22891645

[ref95] El Bakali J.; Gras-Masse H.; Maingot L.; Deprez B.; Dumont J.; Leroux F.; Deprez-Poulain R. Inhibition of aggrecanases as a therapeutic strategy in osteoarthritis. Future Med. Chem. 2014, 6, 1399–1412. 10.4155/fmc.14.84. 25329196

[ref96] Ding Y.; O’Keefe H.; DeLorey J. L.; Israel D. I.; Messer J. A.; Chiu C. H.; Skinner S. R.; Matico R. E.; Murray-Thompson M. F.; Li F.; Clark M. A.; Cuozzo J. W.; Arico-Muendel C.; Morgan B. A. Discovery of potent and selective inhibitors for ADAMTS-4 through DNA-Encoded Library Technology (ELT). ACS Med. Chem. Lett. 2015, 6, 888–893. 10.1021/acsmedchemlett.5b00138. 26288689 PMC4538441

[ref97] Brew K.; Nagase H. The tissue inhibitors of metalloproteinases (TIMPs): an ancient family with structural and functional diversity. Biochim. Biophys. Acta 2010, 1803, 55–71. 10.1016/j.bbamcr.2010.01.003. 20080133 PMC2853873

[ref98] Williamson R. A.; Marston F. A.; Angal S.; Koklitis P.; Panico M.; Morris H. R.; Carne A. F.; Smith B. J.; Harris T. J.; Freedman R. B. Disulphide bond assignment in human tissue inhibitor of metalloproteinases (TIMP). Biochem. J. 1990, 268, 267–274. 10.1042/bj2680267. 2163605 PMC1131427

[ref99] Yu W. H.; Yu S.; Meng Q.; Brew K.; Woessner J. F. Jr. TIMP-3 binds to sulfated glycosaminoglycans of the extracellular matrix. J. Biol. Chem. 2000, 275, 31226–31232. 10.1074/jbc.M000907200. 10900194

[ref100] Logue T.; Lizotte-Waniewski M.; Brew K. Thermodynamic profiles of the interactions of suramin, chondroitin sulfate, and pentosan polysulfate with the inhibitory domain of TIMP-3. FEBS Lett. 2020, 594, 94–103. 10.1002/1873-3468.13556. 31359422

[ref101] Sahebjam S.; Khokha R.; Mort J. S. Increased collagen and aggrecan degradation with age in the joints of Timp3(−/−) mice. Arthritis Rheum. 2007, 56, 905–909. 10.1002/art.22427. 17328064

[ref102] Hashimoto G.; Aoki T.; Nakamura H.; Tanzawa K.; Okada Y. Inhibition of ADAMTS4 (aggrecanase-1) by tissue inhibitors of metalloproteinases (TIMP-1, 2, 3 and 4). FEBS Lett. 2001, 494, 192–195. 10.1016/S0014-5793(01)02323-7. 11311239

[ref103] Kashiwagi M.; Tortorella M.; Nagase H.; Brew K. TIMP-3 is a potent inhibitor of aggrecanase 1 (ADAM-TS4) and aggrecanase 2 (ADAM-TS5). J. Biol. Chem. 2001, 276, 12501–12504. 10.1074/jbc.C000848200. 11278243

[ref104] Wang W. M.; Ge G.; Lim N. H.; Nagase H.; Greenspan D. S. TIMP-3 inhibits the procollagen N-proteinase ADAMTS-2. Biochem. J. 2006, 398, 515–519. 10.1042/BJ20060630. 16771712 PMC1559475

[ref105] Fields G. B. The Rebirth of matrix metalloproteinase inhibitors: moving beyond the dogma. Cells 2019, 8, 984 10.3390/cells8090984. PMC6769477 31461880

[ref106] Scilabra S. D.; Troeberg L.; Yamamoto K.; Emonard H.; Thøgersen I.; Enghild J. J.; Strickland D. K.; Nagase H. Differential regulation of extracellular tissue inhibitor of metalloproteinases-3 levels by cell membrane-bound and shed low density lipoprotein receptor-related protein 1. J. Biol. Chem. 2013, 288, 332–342. 10.1074/jbc.M112.393322. 23166318 PMC3537031

[ref107] Wei S.; Kashiwagi M.; Kota S.; Xie Z.; Nagase H.; Brew K. Reactive site mutations in tissue inhibitor of metalloproteinase-3 disrupt inhibition of matrix metalloproteinases but not tumor necrosis factor-alpha-converting enzyme. J. Biol. Chem. 2005, 280, 32877–32882. 10.1074/jbc.C500220200. 16079149

[ref108] Lim N. H.; Kashiwagi M.; Visse R.; Jones J.; Enghild J. J.; Brew K.; Nagase H. Reactive-site mutants of N-TIMP-3 that selectively inhibit ADAMTS-4 and ADAMTS-5: biological and structural implications. Biochem. J. 2010, 431, 113–122. 10.1042/BJ20100725. 20645923 PMC3003256

[ref109] Kanakis I.; Liu K.; Poulet B.; Javaheri B.; van’t Hof R. J.; Pitsillides A. A.; Bou-Gharios G. Targeted inhibition of aggrecanases prevents articular cartilage degradation and augments bone mass in the STR/Ort mouse model of spontaneous osteoarthritis. Arthritis Rheumatol. 2019, 71, 571–582. 10.1002/art.40765. 30379418

[ref110] Nakamura H.; Vo P.; Kanakis I.; Liu K.; Bou-Gharios G. Aggrecanase-selective tissue inhibitor of metalloproteinase-3 (TIMP3) protects articular cartilage in a surgical mouse model of osteoarthritis. Sci. Rep. 2020, 10, 9288 10.1038/s41598-020-66233-0. 32518385 PMC7283274

[ref111] Doherty C. M.; Visse R.; Dinakarpandian D.; Strickland D. K.; Nagase H.; Troeberg L. Engineered tissue inhibitor of metalloproteinases-3 variants resistant to endocytosis have prolonged chondroprotective activity. J. Biol. Chem. 2016, 291, 22160–22172. 10.1074/jbc.M116.733261. 27582494 PMC5063997

[ref112] Fisher C.; Beglova N.; Blacklow S. C. Structure of an LDLR-RAP complex reveals a general mode for ligand recognition by lipoprotein receptors. Mol. Cell 2006, 22, 277–283. 10.1016/j.molcel.2006.02.021. 16630895

[ref113] Chintalgattu V.; Greenberg J.; Singh S.; Chiueh V.; Gilbert A.; O’Neill J. W.; Smith S.; Jackson S.; Khakoo A. Y.; Lee T. Utility of glycosylated TIMP3 molecules: inhibition of MMPs and TACE to improve cardiac function in rat myocardial infarct model. Pharmacol Res. Perspect. 2018, 6, e00442 10.1002/prp2.442. 30459952 PMC6234480

[ref114] Graham R. C. Jr; Karnovsky M. J. Glomerular permeability. Ultrastructural cytochemical studies using peroxidases as protein tracers. J. Exp. Med. 1966, 124, 1123–1134. 10.1084/jem.124.6.1123. 5925318 PMC2138332

[ref115] Vaccaro C.; Zhou J.; Ober R. J.; Ward E. S. Engineering the Fc region of immunoglobulin G to modulate in vivo antibody levels. Nat. Biotechnol. 2005, 23, 1283–1288. 10.1038/nbt1143. 16186811

[ref116] Alberts B. M.; Sacre S. M.; Bush P. G.; Mullen L. M. Engineering of TIMP-3 as a LAP-fusion protein for targeting to sites of inflammation. J. Cell Mol. Med. 2019, 23, 1617–1621. 10.1111/jcmm.14019. 30450736 PMC6349231

[ref117] Fan D.; Kassiri Z. Biology of tissue inhibitor of metalloproteinase 3 (TIMP3), and its therapeutic implications in cardiovascular pathology. Front. Physiol. 2020, 11, 661 10.3389/fphys.2020.00661. 32612540 PMC7308558

[ref118] Eckhouse S. R.; Purcell B. P.; McGarvey J. R.; Lobb D.; Logdon C. B.; Doviak H.; O’Neill J. W.; Shuman J. A.; Novack C. P.; Zellars K. N.; Pettaway S.; Black R. A.; Khakoo A.; Lee T.; Mukherjee R.; Gorman J. H.; Gorman R. C.; Burdick J. A.; Spinale F. G. Local hydrogel release of recombinant TIMP-3 attenuates adverse left ventricular remodeling after experimental myocardial infarction. Sci. Transl. Med. 2014, 6, 223ra21 10.1126/scitranslmed.3007244. PMC4365799 24523321

[ref119] Barlow S. C.; Doviak H.; Jacobs J.; Freeburg L. A.; Perreault P. E.; Zellars K. N.; Moreau K.; Villacreses C. F.; Smith S.; Khakoo A. Y.; Lee T.; Spinale F. G. Intracoronary delivery of recombinant TIMP-3 after myocardial infarction: effects on myocardial remodeling and function. Am. J. Physiol. Heart Circ. Physiol. 2017, 313, H690–H699. 10.1152/ajpheart.00114.2017. 28754718 PMC5668606

[ref120] Camodeca C.; Cuffaro D.; Nuti E.; Rossello A. ADAM metalloproteinases as potential drug targets. Curr. Med. Chem. 2019, 26, 2661–2689. 10.2174/0929867325666180326164104. 29589526

[ref121] Li K.; Tay F. R.; Yiu C. K. Y. The past, present and future perspectives of matrix metalloproteinase inhibitors. Pharmacol. Ther. 2020, 207, 107465 10.1016/j.pharmthera.2019.107465. 31863819

[ref122] Troeberg L.; Fushimi K.; Khokha R.; Emonard H.; Ghosh P.; Nagase H. Calcium pentosan polysulfate is a multifaceted exosite inhibitor of aggrecanases. FASEB J. 2008, 22, 3515–3524. 10.1096/fj.08-112680. 18632849 PMC2537431

[ref123] Jeske W.; Kouta A.; Farooqui A.; Siddiqui F.; Rangnekar V.; Niverthi M.; Laddu R.; Hoppensteadt D.; Iqbal O.; Walenga J.; Fareed J. Bovine mucosal heparins are comparable to porcine mucosal heparin at USP potency adjusted levels. Front. Med. (Lausanne) 2019, 5, 360 10.3389/fmed.2018.00360. 30687709 PMC6333674

[ref124] Arepally G. M.; Padmanabhan A. Heparin-induced thrombocytopenia: a focus on thrombosis. Arterioscler. Thromb. Vasc. Biol. 2021, 41, 141–152. 10.1161/ATVBAHA.120.315445. 33267665 PMC7769912

[ref125] Takizawa M.; Yatabe T.; Okada A.; Chijiiwa M.; Mochizuki S.; Ghosh P.; Okada Y. Calcium pentosan polysulfate directly inhibits enzymatic activity of ADAMTS4 (aggrecanase-1) in osteoarthritic chondrocytes. FEBS Lett. 2008, 582, 2945–2949. 10.1016/j.febslet.2008.07.036. 18671975

[ref126] Troeberg L.; Mulloy B.; Ghosh P.; Lee M. H.; Murphy G.; Nagase H. Pentosan polysulfate increases affinity between ADAMTS-5 and TIMP-3 through formation of an electrostatically driven trimolecular complex. Biochem. J. 2012, 443, 307–315. 10.1042/BJ20112159. 22299597 PMC3369482

[ref127] Ghosh P.; Edelman J.; March L.; Smith M. Effects of pentosan polysulfate in osteoarthritis of the knee: A randomized, double-blind, placebo-controlled pilot study. Curr. Ther Res. Clin. Exp. 2005, 66, 552–571. 10.1016/j.curtheres.2005.12.012. 24678076 PMC3965979

[ref128] Kumagai K.; Shirabe S.; Miyata N.; Murata M.; Yamauchi A.; Kataoka Y.; Niwa M. Sodium pentosan polysulfate resulted in cartilage improvement in knee osteoarthritis--an open clinical trial. BMC Clin. Pharmacol. 2010, 10, 7 10.1186/1472-6904-10-7. 20346179 PMC2873929

[ref129] Sampson M. J.; Kabbani M.; Krishnan R.; Nganga M.; Theodoulou A.; Krishnan J. Improved clinical outcome measures of knee pain and function with concurrent resolution of subchondral Bone Marrow Edema Lesion and joint effusion in an osteoarthritic patient following Pentosan Polysulphate Sodium treatment: a case report. BMC Musculoskelet. Disord. 2017, 18, 396 10.1186/s12891-017-1754-3. 28899386 PMC5596862

[ref130] Cuffaro D.; Nuti E.; Rossello A. An overview of carbohydrate-based carbonic anhydrase inhibitors. J. Enzyme Inhib. Med. Chem. 2020, 35, 1906–1922. 10.1080/14756366.2020.1825409. 33078634 PMC7717713

[ref131] Nuti E.; Cuffaro D.; D’Andrea F.; Rosalia L.; Tepshi L.; Fabbi M.; Carbotti G.; Ferrini S.; Santamaria S.; Camodeca C.; Ciccone L.; Orlandini E.; Nencetti S.; Stura E. A.; Dive V.; Rossello A. Sugar-based arylsulfonamide carboxylates as selective and water-soluble matrix metalloproteinase-12 Inhibitors. ChemMedChem 2016, 11, 1626–1637. 10.1002/cmdc.201600235. 27356908

[ref132] Cuffaro D.; Camodeca C.; D’Andrea F.; Piragine E.; Testai L.; Calderone V.; Orlandini E.; Nuti E.; Rossello A. Matrix metalloproteinase-12 inhibitors: synthesis, structure-activity relationships and intestinal absorption of novel sugar-based biphenylsulfonamide carboxylates. Bioorg. Med. Chem. 2018, 26, 5804–5815. 10.1016/j.bmc.2018.10.024. 30429099

[ref133] Vankemmelbeke M. N.; Jones G. C.; Fowles C.; Ilic M. Z.; Handley C. J.; Day A. J.; Knight C. G.; Mort J. S.; Buttle D. J. Selective inhibition of ADAMTS-1, −4 and −5 by catechin gallate esters. Eur. J. Biochem. 2003, 270, 2394–2403. 10.1046/j.1432-1033.2003.03607.x. 12755694

[ref134] Cudic M.; Burstein G. D.; Fields G. B.; Lauer-Fields J. Analysis of flavonoid-based pharmacophores that inhibit aggrecanases (ADAMTS-4 and ADAMTS-5) and matrix metalloproteinases through the use of topologically constrained peptide substrates. Chem. Biol. Drug Des. 2009, 74, 473–482. 10.1111/j.1747-0285.2009.00885.x. 19793184 PMC2782546

[ref135] Moncada-Pazos A.; Obaya A. J.; Viloria C. G.; López-Otín C.; Cal S. The nutraceutical flavonoid luteolin inhibits ADAMTS-4 and ADAMTS-5 aggrecanase activities. J. Mol. Med. (Berlin) 2011, 89, 611–619. 10.1007/s00109-011-0741-7. 21365186

[ref136] Zhou J.; Rossi J. Aptamers as targeted therapeutics: current potential and challenges. Nat. Rev. Drug Discovery 2017, 16, 181–202. 10.1038/nrd.2016.199. 27807347 PMC5700751

[ref137] Shu Y.; Pi F.; Sharma A.; Rajabi M.; Haque F.; Shu D.; Leggas M.; Evers B. M.; Guo P. Stable RNA nanoparticles as potential new generation drugs for cancer therapy. Adv. Drug Deliv. Rev. 2014, 66, 74–89. 10.1016/j.addr.2013.11.006. 24270010 PMC3955949

[ref138] Yu Y.; Liu M.; Choi V. N. T.; Cheung Y. W.; Tanner J. A. Selection and characterization of DNA aptamers inhibiting a druggable target of osteoarthritis, ADAMTS-5. Biochimie 2022, 201, 168–176. 10.1016/j.biochi.2022.06.001. 35700850

[ref139] Pluda S.; Mazzocato Y.; Angelini A. Peptide-based inhibitors of ADAM and ADAMTS metalloproteinases. Front. Mol. Biosci. 2021, 8, 703715 10.3389/fmolb.2021.703715. 34368231 PMC8335159

[ref140] Tortorella M.; Pratta M.; Liu R. Q.; Abbaszade I.; Ross H.; Burn T.; Arner E. The thrombospondin motif of aggrecanase-1 (ADAMTS-4) is critical for aggrecan substrate recognition and cleavage. J. Biol. Chem. 2000, 275, 25791–25797. 10.1074/jbc.M001065200. 10827174

[ref141] Hills R.; Mazzarella R.; Fok K.; Liu M.; Nemirovskiy O.; Leone J.; Zack M. D.; Arner E. C.; Viswanathan M.; Abujoub A.; Muruganandam A.; Sexton D. J.; Bassill G. J.; Sato A. K.; Malfait A. M.; Tortorella M. D. Identification of an ADAMTS-4 cleavage motif using phage display leads to the development of fluorogenic peptide substrates and reveals matrilin-3 as a novel substrate. J. Biol. Chem. 2007, 282, 11101–11109. 10.1074/jbc.M611588200. 17311924

[ref142] Zhang W.; Zhong B.; Zhang C.; Wang Y.; Guo S.; Luo C.; Zhan Y. Structural modeling of osteoarthritis ADAMTS4 complex with its cognate inhibitory protein TIMP3 and rational derivation of cyclic peptide inhibitors from the complex interface to target ADAMTS4. Bioorg. Chem. 2018, 76, 13–22. 10.1016/j.bioorg.2017.10.017. 29102725

[ref143] Mullard A. FDA approves 100th monoclonal antibody product. Nat. Rev. Drug Discovery 2021, 20, 491–495. 10.1038/d41573-021-00079-7. 33953368

[ref144] Köhler G.; Milstein C. Continuous cultures of fused cells secreting antibody of predefined specificity. Nature 1975, 256, 495–497. 10.1038/256495a0. 1172191

[ref145] Alfaleh M. A.; Alsaab H. O.; Mahmoud A. B.; Alkayyal A. A.; Jones M. L.; Mahler S. M.; Hashem A. M. Phage display derived monoclonal antibodies: from bench to bedside. Front. Immunol. 2020, 11, 1986 10.3389/fimmu.2020.01986. 32983137 PMC7485114

[ref146] Santamaria S.; de Groot R. Monoclonal antibodies against metzincin targets. Br. J. Pharmacol. 2019, 176, 52–66. 10.1111/bph.14186. 29488211 PMC6284333

[ref147] Chiusaroli R.; Visentini M.; Galimberti C.; Casseler C.; Mennuni L.; Covaceuszach S.; Lanza M.; Ugolini G.; Caselli G.; Rovati L. C.; Visintin M. Targeting of ADAMTS5′s ancillary domain with the recombinant mAb CRB0017 ameliorates disease progression in a spontaneous murine model of osteoarthritis. Osteoarthritis Cartilage 2013, 21, 1807–1810. 10.1016/j.joca.2013.08.015. 23954517

[ref148] Shiraishi A.; Mochizuki S.; Miyakoshi A.; Kojoh K.; Okada Y. Development of human neutralizing antibody to ADAMTS4 (aggrecanase-1) and ADAMTS5 (aggrecanase-2). Biochem. Biophys. Res. Commun. 2016, 469, 62–69. 10.1016/j.bbrc.2015.11.072. 26612259

[ref149] Larkin J.; Lohr T.; Elefante L.; Shearin J.; Matico R.; Su J. L.; Xue Y.; Liu F.; Rossman E. I.; Renninger J.; Wu X.; Abberley L.; Miller R. E.; Foulcer S.; Chaudhary K. W.; Genell C.; Murphy D.; Tran P. B.; Apte S.; Malfait A. M.; Maier C. C.; Matheny C. J. The highs and lows of translational drug development: antibody mediated inhibition of ADAMTS-5 for osteoarthritis disease modification. Osteoarthritis Cartilage 2014, 22, S483 10.1016/j.joca.2014.02.918. PMC4516626 25800415

[ref150] Siebuhr A. S.; Werkmann D.; Bay-Jensen A. C.; Thudium C. S.; Karsdal M. A.; Serruys B.; Ladel C.; Michaelis M.; Lindemann S. The anti-ADAMTS-5 nanobody^®^ M6495 protects cartilage degradation ex vivo. Int. J. Mol. Sci. 2020, 21, 5992 10.3390/ijms21175992. PMC7503673 32825512

[ref151] Sharma N.; Drobinski P.; Kayed A.; Chen Z.; Kjelgaard-Petersen C. F.; Gantzel T.; Karsdal M. A.; Michaelis M.; Ladel C.; Bay-Jensen A. C.; Lindemann S.; Thudium C. S. Inflammation and joint destruction may be linked to the generation of cartilage metabolites of ADAMTS-5 through activation of toll-like receptors. Osteoarthritis Cartilage 2020, 28, 658–668. 10.1016/j.joca.2019.11.002. 31734268

[ref152] Miller R. E.; Ishihara S.; Tran P. B.; Golub S. B.; Last K.; Miller R. J.; Fosang A. J.; Malfait A. M. An aggrecan fragment drives osteoarthritis pain through Toll-like receptor 2. JCI Insight 2018, 3, e95704 10.1172/jci.insight.95704. PMC5926921 29563338

[ref153] Guehring H.; Goteti K.; Sonne J.; Ladel C.; Ona V.; Moreau F.; Bay-Jensen A.-C.; Bihlet A. R. Safety, tolerability, pharmacokinetics and pharmacodynamics of single ascending doses of the anti-ADAMTS-5 nanobody, M6495, in healthy male subjects: a phase I, placebo-controlled, first-in-human study. Arthritis Rheumatol. 2019, 71, 3826–3829. 10.1002/central/CN-02195478.

[ref154] Alves-Simões M. Rodent models of knee osteoarthritis for pain research. Osteoarthritis Cartilage 2022, 30, 802–814. 10.1016/j.joca.2022.01.010. 35139423

[ref155] Le Graverand-Gastineau M.-P. H. OA clinical trials: current targets and trials for OA. Choosing molecular targets: what have we learned and where we are headed?. Osteoarthritis Cartilage 2009, 17, 1393–1401. 10.1016/j.joca.2009.04.009. 19426849

[ref156] Kim Y.; Levin G.; Nikolov N. P.; Abugov R.; Rothwell R. Concept end points informing design considerations for confirmatory clinical trials in osteoarthritis. Arthritis Care Res. (Hoboken) 2022, 74, 1154–1162. 10.1002/acr.24549. 33345469

[ref158] Koch C. D.; Lee C. M.; Apte S. S. Aggrecan in cardiovascular development and disease. J. Histochem. Cytochem. 2020, 68, 777–795. 10.1369/0022155420952902. 32870742 PMC7649964

[ref159] Wang H.; Bai J.; He B.; Hu X.; Liu D. Osteoarthritis and the risk of cardiovascular disease: a meta-analysis of observational studies. Sci. Rep. 2016, 6, 39672 10.1038/srep39672. 28004796 PMC5177921

[ref160] Veronese N.; Cereda E.; Maggi S.; Luchini C.; Solmi M.; Smith T.; Denkinger M.; Hurley M.; Thompson T.; Manzato E.; Sergi G.; Stubbs B. Osteoarthritis and mortality: A prospective cohort study and systematic review with meta-analysis. Semin. Arthritis Rheum. 2016, 46, 160–167. 10.1016/j.semarthrit.2016.04.002. 27179749

[ref161] Velásquez Pereira L. C.; Roose E.; Graça N.; Sinkovits G.; Kangro K.; Joly B. S.; Tellier E.; Kaplanski G.; Falter T.; Von Auer C.; Rossmann H.; Feys H. B.; Reti M.; Prohászka Z.; Lämmle B.; Voorberg J.; Coppo P.; Veyradier A.; De Meyer S. F.; Männik A.; Vanhoorelbeke K. Immunogenic hotspots in the spacer domain of ADAMTS13 in immune-mediated thrombotic thrombocytopenic purpura. J. Thromb. Haemost. 2021, 19, 478–488. 10.1111/jth.15170. 33171004

[ref162] Blouse G. E.; Bøtkjaer K. A.; Deryugina E.; Byszuk A. A.; Jensen J. M.; Mortensen K. K.; Quigley J. P.; Andreasen P. A. A novel mode of intervention with serine protease activity: targeting zymogen activation. J. Biol. Chem. 2009, 284, 4647–4657. 10.1074/jbc.M804922200. 19047064 PMC2640953

[ref163] Garcia-Ferrer I.; Marrero A.; Gomis-Rüth F. X.; Goulas T. α2-macroglobulins: structure and function. Subcell. Biochem. 2017, 83, 149–183. 10.1007/978-3-319-46503-6_6. 28271476

[ref164] Harwood S. L.; Nielsen N. S.; Diep K.; Jensen K. T.; Nielsen P. K.; Yamamoto K.; Enghild J. J. Development of selective protease inhibitors via engineering of the bait region of human α2-macroglobulin. J. Biol. Chem. 2021, 297, 100879 10.1016/j.jbc.2021.100879. 34139236 PMC8267569

[ref165] Santamaria S.; de Groot R. ADAMTS proteases in cardiovascular physiology and disease. Open Biol. 2020, 10, 200333 10.1098/rsob.200333. 33352066 PMC7776578

[ref166] Dupuis L. E.; Nelson E. L.; Hozik B.; Porto S. C.; Rogers-DeCotes A.; Fosang A.; Kern C. B. Adamts5–/– mice exhibit altered aggrecan proteolytic profiles that correlate with ascending aortic anomalies. Arterioscler. Thromb. Vasc. Biol. 2019, 39, 2067–2081. 10.1161/ATVBAHA.119.313077. 31366218 PMC6761016

